# Developmental Changes in the Structure of Executive Function from Early To Late Adolescence

**DOI:** 10.1007/s10964-025-02293-7

**Published:** 2025-12-04

**Authors:** Jianping Ma, Xiaoxi Liu, Teng Pan, Linqin Ji, Wenxin Zhang

**Affiliations:** 1 Faculty of Psychology, Shandong Normal University, Jinan, Shandong Province China; 2 Shandong Provincial Key Laboratory of Brain Science and Mental Health, Jinan, Shandong Province China

**Keywords:** Executive functions, Development, Component structure, Specialization and reorganization

## Abstract

The organization of functional components of executive functions during the critical developmental period of adolescence remains poorly understood. The current study was to examine whether there were age-related differences in component structures of executive function from around age 10 to 18. Using a cohort-sequential design, a sample of 858 Chinese adolescents (grade 5, *n* = 287, *M*_age_ = 10.72, *SD* = 0.39, 41.8% girls; grade 7, *n* = 304, *M*_age_ = 12.74, *SD* = 0.42, 40.8% girls; grade 9, *n* = 267, *M*_age_ = 14.71, *SD* = 0.47, 52.8% girls) were tested for five waves over 4 years (T1, Dec. 2020). Executive function was assessed using eight well-validated cognitive tasks, measuring three core components: inhibition (Flanker, Stroop), switching (Dimensional Change Card Sort, Picture–Symbol, Wisconsin Card Sorting Test), and updating (Backward Digit Span, List Sorting, Visuospatial Memory). Results revealed the task performance improved with age cohorts and timepoints. Confirmatory factor analyses and tests for longitudinal factorial invariance showed that data from younger adolescents (ages 10–11) and older adolescents (ages 15+) fit a two-factor model where inhibition and switching were merged, whereas a well-separated three-factor structure emerged between ages 11–15. Executive functions differentially predicted academic achievement, peer relationships, and internalizing/externalizing symptoms across grade cohorts (G5, G7, G9), with no significant effects on planning performance. The results suggested that the development of executive function is characterized by both specialization and functional integration, challenging the prevailing view of a linear trajectory of increase specialization of executive functions across adolescence.

**Supplementary Information:**

The online version contains supplementary material available at 10.1007/s10964-025-02293-7.

## Introduction

Executive function refers to a set of general-purpose control mechanisms that are linked to the brain’s prefrontal cortex and regulates the dynamics of human cognition and action (Diamond, 2012; Zelazo, 2020). Executive function is crucial for children and adolescents’ academic achievements and interpersonal relationships, while its impairment has been linked to emotional and behavioral problems (Ahmed et al., 2019; Morea & Calvete, 2021; Wang & Zhou, 2019; Zelazo, 2020). There have been consistent debates regarding the component structure of executive function especially for children and adolescents. Both theorizing perspectives (Breit et al., 2021; Kovacs & Conway, 2016) and empirical studies (e.g., Brydges et al., 2014; Friedman & Miyake, 2017; Lee et al., 2013; Miller et al., 2012; Usai et al., 2014; Wu et al., 2011) suggest that executive functions differentiate with age and ability. However, prior studies have important methodology limitations, including underpowered samples, the use of samples encompassing broader age ranges (which may confound the examination of executive function structure across development), and the use of task batteries that did not cover the three principal components of executive functions. Further and more importantly, few studies have examined executive function development around age 10 to 18 (Best & Miller, 2010; Karr et al., 2018, 2022). Despite the rapid improvements in executive functioning ability during early childhood, the prolonged maturation of executive functions and their associated prefrontal circuits continues through late adolescence and emerging adulthood (Best & Miller, 2010; Diamond, 2013; Folker et al., 2025; Zelazo, 2020). Using a cohort-sequential design with a Chinese adolescent sample, the current study was to examine developmental changes in component structure of executive function from early to late adolescence. Elucidating these developmental patterns is essential for understanding how cognitive control systems transition from adolescence to adulthood.

### Component Structure of Executive Function among Children and Adolescence

The component structure is crucial in research on executive function, as it relates to the operationalization and measurement, and directly impacts the inferences researchers can make about these competencies (Diamond, 2013; Miyake et al., 2000; Zelazo, 2020). A leading framework on structure of executive function is the integrative model by Miyake and colleagues (2000, 2004), which employed a heuristic approach and postulated that executive function is a construct of both unity and diversity, and the core components are inhibition, shifting, and updating. Inhibition refers to the ability to deliberately suppress dominant, automatic, or prepotent responses when necessary, switching involves the ability to flexibly transition between different tasks, mental sets, or strategies in response to changing demands, and updating pertains to the active monitoring and modification of working memory representations, allowing individuals to maintain and refresh relevant information while discarding or replacing outdated content (Miyake et al., 2000; Dimond, 2013; Zelazo, 2020). This general pattern of unity and diversity of executive function has been replicated in samples, including young adults (e.g., Friedman et al., 2006; Klauer et al., 2010) and older adults (Fisk & Sharp, 2004; de Frias et al., 2009; Vaughan & Giovanello, 2010). The integrative model is particularly valuable for investigating the development of the executive function structure because it explicitly accommodates developmental changes in both the specialization and reorganization of components. While inhibition has long been linked to regulatory processes central to self-control, the integrative model highlights that flexibility and updating are equally essential for adaptive functioning (Miyake & Friedman, 2012), particularly during adolescence when individuals must coordinate cognitive stability and flexibility in increasingly complex social and academic contexts. This broader perspective situates executive function as a dynamic, multi-faceted construct that undergoes differentiation and reorganization across development, thereby offering a more comprehensive framework for understanding cognitive maturation than approaches that emphasize self-control alone. Furthermore, the novelty of the integrative model lay in two key elements: the adoption of a confirmatory approach to test a priori specified models of executive function, and the utilization of a sufficient number of tests to measure each of the three hypothesized core components (Diamond, 2013).

Executive function emerges during the first few years of life, and continues to strengthen significantly throughout childhood and adolescence (Best & Miller, 2010). Theorizing perspectives suggest developmental differentiation in component structure of executive function, that is, factor structure changes from a strongly general ability to separable correlated abilities (Breit et al., 2021; Kovacs & Conway, 2016). The developmental differentiation of executive function ability parallels the increasing functional specialization and modularity of neural systems in the developmental process of adaptions (see Fiske & Holmboe, 2019 for systematic review), as theorized in the interactive specialization model (Johnson & Munakata, 2005). Empirical studies provided converging evidence for age variations in executive function structure across childhood to adolescence (Karr et al., 2018). Studies identified a unitary factor model during early childhood (e.g., Brydges et al., 2014; Masten et al., 2012), and two- or three-factor models among school-age children and adolescents (e.g., Friedman et al., 2016; Klauer et al., 2010; Lee et al., 2013; Miyake et al., 2000; Wu et al., 2011; Xu et al., 2013). Although, there were also contradictory research findings (Brocki & Bohlin, 2004; Huizinga et al., 2006; McAuley & White, 2011).

However, extant studies on the component structure of executive function across development possess important methodology shortcomings, including the use of executive function tests where the principal component measured was unclear, use of a battery of tests that failed to cover the three principal components, inclusion non-executive function measure, and underpowered samples (Best & Miller, 2010; Karr et al., 2018, 2022). Moreover, and more critically, few studies have systematically investigated the development and component structure of executive functions across the period spanning early to late adolescence. While some prior research has tapped the component structure of executive function for certain ages during this developmental phase, and identified a one- or two-factor structure in late childhood and early adolescence, with a shift toward a three-factor model emerging by middle to late adolescence (e.g., 11–15 years old; Lee et al., 2013; Xu et al., 2013), comprehensive longitudinal analyses remain scarce around age 10 to 18.

Adolescence is a particularly interesting time for executive function development. Studies suggested that differentiation development in inhibition, shifting, and updating occurs at around the early adolescent years, with adult-level proficiency typically achieved by late adolescence (e.g., Luciana et al., 2005; Chaku & Hoyt, 2019; Folker et al., 2025). These performance improvements coincide with synaptic pruning and myelination throughout the brain (Zelazo, 2020), and a barrage of new environmental influences and transitions that occurs in classroom and schools, families, and the social contexts (Steinberg, 2023). Accordingly, there may be new developmental features in the component structure of executive function since early to late adolescence.

A theoretical issue complicating the component framework of executive function during adolescence revolves around the structural relationship between inhibition and shifting (Dreisbach et al., 2024; Egner, 2023; Hommel et al., 2024). Despite studies posited that these constructs are conceptually and empirically distinguishable in adult-level executive function (Egner, 2023; Miyake et al., 2000), emerging perspectives argued that inhibition and shifting are not fully independent processes but rather two facets of a unified dynamic control system (Dreisbach et al., 2024; Hommel et al., 2024). Crucially, the recent theoretical accounts posited that task focus and task switch reflect overlapping mechanisms within a broader adaptive control system when the adult-level cognitive control capacities develop.

### Cultural Consideration

China possesses distinct cultural and societal features. To be specific, Chinese traditional culture put great emphasis on self-control (Kitayama & Salvador, 2024). In the Chinese family and school context, parents and teachers tend to expect impulse control among children, and constantly implement self-control and self-regulation practice on children, by giving substantially more proactive self-regulatory instructions, such as “pay attention” ‘‘do properly’’ and ‘‘avoid doing something’’ (Ng et al., 2014). Accordingly, Chinese children may have many culturally defined opportunities to exercise and practice executive function skills. Studies that compared the executive function among Chinese children and those in American and other western countries tend to reported higher scores in performance on inhibition and attentional control tasks (e.g., Ng et al., 2014). Similarly, Xu et al. (2020) replicated the component structure on both Western and Eastern samples; they found that Western perform lower in executive function than Eastern culture.

### The Prediction of Executive Function on Planning and Psychosocial Adjustments

Executive cognitive functions were involved in complex tasks and success in daily life. First, the components such as inhibition, updating, and shifting have been viewed as building blocks for more complex executive function, such as planning (Miyake et al., 2000). Planning is frequently described as an ability that involves mapping out a sequence of moves in preparation for the task, including inhibiting dominant or habitual responses, updating information, and switching between multiple tasks. Second, executive function was involved during the solution of important daily complex tasks. It has been found that executive function was associated with good academic performance and peer relationships, and conversely executive function deficits affect and impair academic performance and good interpersonal relationships (Ahmed et al., 2019; Wang & Zhou, 2019). Children need to resist distraction to focus on teachers’ instruction, to hold and manipulate relevant information in working memory, and to mentally represent and plan problem-solving strategies, all are implicated in executive function. Complex executive function skills that require the monitoring of overt, deliberate activities are particularly useful in a learning environment where students are constantly expected to pay attention, follow rules, and concentrate on various cognitive and behavioral tasks (Ahmed et al., 2019). Further, to engage in adaptive peer interaction, children need to inhibit inappropriate emotion, response, and behavior (e.g., being aggressive and losing temper), all rely on executive function (Zelazo, 2020). Third, executive function deficits have been consistently reported to be involved in internalizing and externalizing problems, due to poor regulation of cognition, emotion, and behaviors, and the deteriorating impacts on academic and peer relationships (Yang et al., 2022; Zelazo, 2020).

## Current Study

Despite the centrality of adolescence for neurocognitive maturation, research has not yet provided a coherent developmental account of how the structure of executive function changes from early to late adolescence. Most existing work has focused on childhood or isolated age groups, leaving uncertain when the three core components—inhibition, shifting, and updating—become fully differentiated or whether they later reorganize into more integrated functional systems. The scarcity of longitudinal studies that simultaneously assess all three components across this developmental window has limited the field’s ability to characterize the timing and mechanisms of structural change. To address these limitations, this study used a cohort-sequential design to examine developmental changes in component structure of executive function from early (around 10-year-olds) to late adolescence (around 18-year-olds). Adolescents were recruited into three age-based cohorts, 2 years apart, and were tested generally at an interval of half a year for four years. Based on the theoretical perspectives and previous findings of differentiation since childhood to adolescent years, the study hypothesized a general differentiation trend in component structures of executive function beginning in early adolescence to middle and late adolescent years. Whereas, given the debate over the structure of cognitive stability and flexibility, potential reorganization and integration of executive functions—specifically the interplay and reciprocity between inhibition and switching—during the late adolescence remains an open empirical question. No specific hypotheses or expectations are made regarding this potential developmental process. The study expected that the executive functions significantly predicted outcomes, whereas the effects differed across components and domain of adjustment.

## Methods

### Participants and Procedures

Data were from the ongoing Multi-cohort Longitudinal Study of Chinese Children and Adolescents (McLSCCA). Participants were 858 adolescents from grade 5 (*M*_age_ = 10.72, *SD* = 0.39, 167 boys, 120 girls), grade 7 (*M*_age_ = 12.74, *SD* = 0.42, 180 boys, 124 girls), and grade 9 (*M*_age_ = 14.71, *SD* = 0.47, 126 boys, 141 girls) in one primary and four junior schools in east China. With following up, the participants were distributed across nine schools (four junior and five senior schools).

In McLSCCA, children and adolescents were generally tested twice per year over four years. Five waves (T1, December 2020; T2, June 2021; T3, December 2021; T4, June 2022; T5, June 2023) of data were used for this study. No data was collected in December 2022, due to the COVID-19 pandemic, leaving the last two-time one year apart. At T4, students from one participating school did not participate the data collection because that the school was not able to schedule time for it. As a result, only 202 participants from the second cohort completed the T4 measure. Due to grade progression, participants in the third cohort (Grade 9) suffered great attrition in the transition from junior school (T2) to senior school (T3), resulting in 196 participants at T3 and since on. Participants in the first cohort (Grade 5) suffered great attrition in the transition from primary school (T4) to junior middle school (T5), resulting in 267 participants at T5.

Of the 858 participants, the proportions who completed each wave were as follows: T1, 78.3% (*n* = 672); T2, 92.4% (*n* = 793); T3, 84.5% (*n* = 725); T4, 76.8% (*n* = 659); T5, 82.5% (*n* = 708). The average age of parents was 39.8 ± 6.28 years for mothers and 40.1 ± 5.67 years for fathers. In terms of educational attainment, 20.2% of mothers and 11.0% of fathers had completed elementary school or below, while junior high school was the most common level, representing 45.7% of mothers and 48.0% of fathers. High school education was attained by 20.4% of mothers and 24.9% of fathers, and 13.4% of mothers and 15.5% of fathers held a college degree and higher. The parents’ education levels in the sample were broadly aligned with those reported in China’s 2020 national population census (National Bureau of Statistics, & P. R., China, 2021). Information of the demographics characteristics was presented in Table S1 in supplementary.

Participants completed tasks (8 tasks for inhibition, switching, and updating, and one task for planning) on computers located in a school computer room. The executive function tasks selected for this study have been widely validated for use with children and adolescents. Specifically, the Flanker and Stroop tasks have been used extensively as measure of inhibition with children and adolescent as 5 years to 18 years (Karr et al., 2022; Lee et al., 2013; Wu et al., 2011; Zelazo, 2020), and the Picture–Symbol, Dimensional Change Card Sort (DCCS), and Wisconsin Card Sorting Test (WCST) are the most established measures of cognitive flexibility in developmental research (Karr et al., 2022; Zelazo, 2020). Likewise, the Backward Digit Span, 2-List List Sorting, and Visuospatial Memory Task tasks have shown reliable age-related variation across childhood and adolescence (Karr et al., 2022; Miyake et al., 2001; Tulsky et al., 2013, 2014). Prior studies employing similar batteries have documented meaningful differentiation of executive function components in samples spanning ages 6 to 18 (e.g., Lee et al., 2013; Xu et al., 2013).

The complete set of tasks was divided into three separate sessions over a one-week period. The total testing time (approximately 90 min) was thereby distributed to alleviate cognitive fatigue and enhance data quality. Within each session, a 2-minute break was incorporated after every task to help maintain focus and performance. Besides, information on academic performance was obtained by school records, peer relationships were assessed through peer nomination in classrooms, and externalizing and internalizing problems were assessed through self-report. Participants received a small gift upon finishing the questionnaires and tasks. Written informed consents were obtained from participants and their parents prior to the study. The study was approved by the Human Subjects Research Ethics Committee in the Department of Psychology of Shandong Normal University.

### Measure

#### Inhibition

 Two computerized tasks, i.e., Flanker task (Eriksen & Eriksen, 1974) and Stroop color-word interference task (Tillman & Wiens, 2011) were used to assess efficiency in inhibition. In the Flanker task (Lee et al., 2013), the children were presented with a row of five fish facing either left or right on a computer screen, with the target fish positioned at the center. The target fish was flanked on either side by two fish facing the same or the opposite direction (congruent or incongruent conditions, respectively). In each trial, participants were asked to identify, by key press, the direction of the target fish. In the Stroop task, children were presented with color words (“红色(red)” and “绿色(green)”) printed in either the congruent ink color (e.g., “红色(red)” in red), or incongruent color (i.e., “红色(red)” in green). Children were instructed to respond to the ink color but not the word meaning, by pressing one of two keys (← for “red”, and → for “green”) on a regular keyboard.

In both tasks, the formal experiment included 20 congruent and 20 incongruent trials which were randomly presented. In line with the definition of inhibition referring to the ability to resist interference from competing or prepotent responses or processes, this study chose the number of correct trials in incongruent condition as an indicator of inhibition.

#### Switching

 Three tasks were used to assess children’s switching ability. A modified version of the Dimensional Change Card Sort (Frye et al., 1995; Hanania, 2010) task was used, with an added third stage. This task included target cards (e.g., red and blue rabbits and boat) and test cards (e.g., green and yellow flowers and cars). The DCCS task included three phases: pre-switch (sorting by the initial rule, e.g., color), post-switch (sorting by the new rule, e.g., shape), and mixed (switching between rules as instructed). Participants responded to the rules by pressing one of two keys (←, →) on a regular keyboard. The present study used the classic DCCS task as practice trials and the third stage as the formal experiment.

As for Picture–Symbol task (Miyake et al., 2000), in each trial, a bigram consisting of a picture and a symbol appeared in one of four quadrants on the computer screen. When the bigram appeared in the top quadrants, children identified whether the picture depicted an animal; when it appeared in the bottom quadrants, they judged the number’s parity (odd/even). They responded to the pictures by pressing one of two keys (“←” = animal/odd, “→” = car/even) on a regular keyboard. The task comprised four blocks: an 21-trial block with bigrams in top quadrants only, a 21-trial block with bigrams in bottom quadrants only, and two final 33-trial blocks with bigrams in all quadrants rotating clockwise. Presentations of the blocks were counterbalanced. This study used the number of correct trials in the conditional switches (i.e., animal to number, or number to animal) in the difficult mixed blocks as the indicator of shifting. 

In the Wisconsin Card Sorting Test (WCST; Heaton & Staff, 1993), children matched individually presented target cards (center screen) to one of four reference cards (top screen) based on three possible attributes: color (red/green/blue/yellow), number (1/2/3/4), or shape (circle/cross/star/square). They responded by pressing a corresponding key (1–4) on a standard keyboard. Each target card remained visible for up to 10s or until a response, followed by visual feedback (“RIGHT”/“WRONG”) and immediate transition to the next trial. The sorting criterion (e.g., color) persisted until 10 consecutive correct sorts, after which it shifted (e.g., to number). Children were informed of criterion changes but not the 10-trial requirement. The task terminated after 64 trials.

In the DCCS and Picture–Symbol task number of correct switch trials was computed from trials involving conditional switches (i.e., “animal” to “number”, “shape” to “color”). In this study, the total number of switches performed by individuals was not consistent. This study computed the ratio of the number of correct switches to the total number of switch as the indicator of DCCS and Picture–Symbol task. For the WCST task, this study chose the number of categories (Huizinga et al., 2006) as the switching index.

#### Updating

 This study used three tasks to assess the updating capacity. In a modified version of the Backwards Digit Span Task (BDST; Drachman & Ommaya, 1964) in which this study used animals to replace the digits, children inversely repeated the sequence of animals presented on the screen for 2s. The number of animals increased by one after each correct response until the child failed twice at the same length or the number of successfully repeated animals reached seven. The participants reported the sequent (inversed) of animals verbally. This study recorded their report and used the highest number of correctly recalled animals in sequence as the indicator of updating ability.

In the 2-List List Sorting (Tulsky et al., 2013, 2014), two categories (i.e., animal or food) were visually presented on the computer screen for 2s with their name simultaneously displayed below. The children sorted the stimuli by size within each category (the category order is fixed, with food first followed by animals). The task starts with a two-item sequence and progressively increases by one item per trial, up to a maximum of seven items. The task terminated when either the child made errors on two trials of the same sequence length, or all seven-item sequences were correctly completed. This study used the highest number of correctly recalled items in sequence as the indicator of updating ability.

In the Visuospatial Memory Task (Miyake et al., 2001), child recalled the sequence of dots appeared one by one on the screen in the 16 grids of 4 × 4. The child responded by clicking with the mouse the blank grid in order as soon as possible. The number of dots started with two and increased by one after each successful trial, continuing until either the child successfully completed the eight-dot memory task, or two consecutive failures occurred. This study used the highest number of dots correctly recalled in sequence as an indicator.

#### Planning

 The computerized version of the Tower of Hanoi (HOT) task (Miyake et al., 2000) was used. Specifically, the child moved from the starting configuration consisting of four disks of varying sizes positioned on three pegs to the ending configuration (presented to the child at the beginning of the task). The children were instructed to minimize both the number of moves and the time necessary to accomplish the reconfiguration. When moving the disks, the children followed a set of rules commonly imposed on the TOH task. This study used the number of moves taken to complete the task as indicator of planning.

#### Academic performance

 Following previous studies, information concerning achievement (Chinese, Math, and English) was obtained from school records at each wave. Given the difference in exam contents across grades and classes, raw scores on the three subjects were standardized within each class and then averaged to form a single index of achievement (Zhang et al., 2019).

#### Peer rejection and acceptance

 The data on peer rejection (“*In your class*,* who do you like least to play with*”) and peer acceptance (“*In your class*,* who do you like to play with most*”) were assessed through procedure of peer nomination, with both same-sex and cross-sex nominations being allowed. The negative and positive nominations each child received were summed separately, and then standardized within each class.

#### Internalizing and externalizing symptomatology

 Internalizing and externalizing symptomatology were assessed with the Youth Self-Report of Child Behavior Checklist (CBCL-YSR; Achenbach & Rescorla, 2001), on a 3-point scale ranging from “0 = not true” to “2 = very true.” Raw mean scores from the internalizing symptomatology (depression/anxiety, 13 items; e.g., “*worries*”, and depression/withdrawal, 5 items; e.g., “*prefer alone*”) and externalizing symptomatology (aggression, 9 items; e.g., “*argues*”, and rule-breaking, 5 items; e.g., “*run away*”) scales were used, with higher scores indicating more severe symptoms. The McDonald’s *ω*s for internalizing from T1 to T5 were 0.92, 0.93, 0.94, 0.93, and 0.93, and for externalizing from T1 to T5 were 0.85,0.86, 0.87, 0.87, and 0.87, respectively.

### Statistical Analysis

For the inhibition and switching tasks, trials with reaction times < 200 ms were automatically excluded. Following previous studies (Friedman & Miyake, 2017), this study applied appropriate trimming and transformation to the task data to improve the distributions and reduce the influence of outliers. First, data trimming was conducted on the inhibition tasks that required key-press responses. Further, if the accuracy of performance was less than 55% in one of the conditions in Inhibition tasks, the results from this task were coded as missing (Huizinga et al., 2006). These procedures resulted in missing rates of 0.7% **–** 3.2% in Flanker, and 2.9% **–** 7.4% in Stroop. Accuracy was high for both tasks (Flanker, 90% to 96%; Stroop, 86% to 95%). Second, for each measure, values exceeding three standard deviations from the group mean were winsorized and replaced with values corresponding to three standard deviations above or below the mean, as appropriate. This approach retained participants’ rank order while mitigating the influence of extreme outliers on correlations and model parameters. Specifically, 3.0% -- 4.3% of Flanker, 2.3% -- 4.0% of Stroop, 1.5% -- 3.5% of DCCS, 0% -- 0.2% of Picture–Symbol task, 0% of WCST, 0.6% -- 0.7% of Visuospatial Memory Task, 0.1% -- 1.4% of 2-List List Sorting, and 0% -- 1.7% of BDST, were replaced with mean ± 3 *SD*s as the highest or lowest scores. Through these procedures, all variables became more normal (-2.83 to 0.36 for the skewness, -0.79–6.95 for the kurtosis).

Little’s test of Missing Completely at Random (MCAR), χ^2^ = 8483.76, *df* = 6920, *p* < .001, indicated that the data were not missing completely at random. For adolescents with complete data and those with missing data, there were no significant differences in terms of gender (χ^2^ [1] = 0.68, *p* = .352), performance on executive function tasks (*t*s= -1.75–1.50, *p*s > 0.05, except for BDST at T1, T2, and T5, *t*s ≥ 2.05, *p*s < 0.05); adolescents participating at all five measurement waves were slightly younger than those with missing data (initial age, *t*[798.42] = 9.45, *p* < .001, *d* = 0.62). The Full Information Maximum Likelihood estimation was performed, which is a robust method embedded within the structural modelling framework that uses all available data to provide the most efficient and least biased estimates under the missing at random assumption (Muthén & Muthén, 2017).

Data analyses proceeded in three phases. First, the study conducted a series of repeated-measure ANOVAs on performance of executive function tasks (the correct numbers or max-level of performance depending on tasks) based on a 5 (waves: timing of data collection) × 3 (cohort: G5, G7, and G9) × 2 (gender: boy and girl) split-plot design. Second, to examine how the component structure of executive function changed across development, the study conducted a series of confirmatory factor analyses to test and compare the one- (a single-factor model with all tasks loading on it), two- (all possible two-factor combinations: Model 2a: inhibition/switching + updating; Model 2b: inhibition/updating + switching; Model 2c: inhibition + switching/updating), and three-dimensional (Inhibition, Switching, Updating) structures of executive function within each age cohort at each time point (see Fig. S1 for a visual representation of each model). Through the framework of confirmatory factor analysis, the observed variance of each task is partitioned into the portion explained by the specified latent factors (executive function components) and the residual, task-unique portion (task-specific variance). Third, the study examined the criterion validity of the component structures by testing the prediction of executive function components on planning, academic performance, peer rejection and acceptance, and externalizing and internalizing problems. In the second step, model competitions followed the procedure suggested by Monette et al. (2015). All confirmatory factor analyses were conducted in Mplus using maximum likelihood estimation with robust standard errors (MLR). Following guidelines by Little (2013), this estimator accounts for potential non-normality and yields robust fit indices. Model comparisons were evaluated using a combination of fit indices (χ²/*df* ≤ 5, CFI ≥ 0.90, TLI ≥ 0.90, RMSEA ≤ 0.08, SRMR ≤ 0.08) and changes in comparative fit indices (ΔCFI ≥ 0.01; ΔRMSEA ≥ 0.015). When two models showed comparable fit, the more parsimonious model was preferred.

## Results

### Preliminary Analyses

Performance scores ranged from 0 to 20 for the Flanker and Stroop tasks, from 0 to 1 for the DCCS and Picture–Symbol task, from 0 to 5 for the WCST, from 1 to 9 for the Visuospatial Memory Task, and from 0 to 7 for both the BDST and the 2-List List Sorting. Significant positive correlations were observed among most cognitive measures (expect for updating index), and correlations between measures of the same construct tended to be moderate (see Table S2). These patterns suggest robust convergent validity among executive function tasks. Across time points and cohorts, adolescents’ cognitive task performance generally improved. The correlation between inhibition and shifting is higher than that between inhibition and updating, and between shifting and updating. However, the correlation between cognitive measures declined over waves. A graphical depiction of the performance scores was provided in Fig. S2.

Results of repeated-measure ANOVAs indicated that, across all eight tasks significant grade cohort differences were found (*F*s ≥ 5.04, *p*s < 0.01), suggesting that performance scores increase from G5 to G9. For tasks that originally were designed to measure shifting and updating (i.e., Picture–Symbol task, DCCS, WCST, Visuospatial Memory Task, 2-List List Sorting, and BDST) there were significant differences across some waves, which generally reflected an increase in performance scores with advancing wave level. These results generally reflected that behavioral performance significantly improved with age and overtime. Further, for Flanker (*F* (1, 422) = 7.51, *p* = .006, $\:{{\upeta\:}}_{p}^{2}$ = 0.02), Stroop (*F* (1, 367) = 5.66, *p* = .018, $\:{{\upeta\:}}_{p}^{2}$ = 0.02), Picture–Symbol task (*F* (1, 434) = 9.95, *p* = .002, $\:{{\upeta\:}}_{p}^{2}$ = 0.02), and WCST (*F* (1, 413) = 23.86, *p* < .001, $\:{{\upeta\:}}_{p}^{2}$ = 0.05) task, boys scored lower than girls; for Visuospatial Memory Task, boys scored higher than girls; no significant gender differences were found for DCCS (*F* (1, 424) = 1.85, *p* = .175, $\:{{\upeta\:}}_{p}^{2}$ = 0.004), 2-List List Sorting (*F* (1, 364) = 1.50, *p* = .22, $\:{{\upeta\:}}_{p}^{2}$ = 0.004), and BDST (*F* (1, 365) = 0.01, *p* = .93, $\:{{\upeta\:}}_{p}^{2}$ = 0.000) task. Detailed descriptions of the results of the ANOVAs were presented in supplementary (see Table S3).

To examine the possible performance differences between administration mode (individual vs. group), the study computed standardized scores for each task and calculated the average performance across the six group-administered (inhibition, switching, and one updating) tasks and the two individually administered (updating) tasks for each participant. A paired-sample t test revealed no significant difference between individual and group administration modes, ts= − 0.522 − 0.177, *p* > .05 (See Table S4).

### Factor Structure

**Fig. 1:**
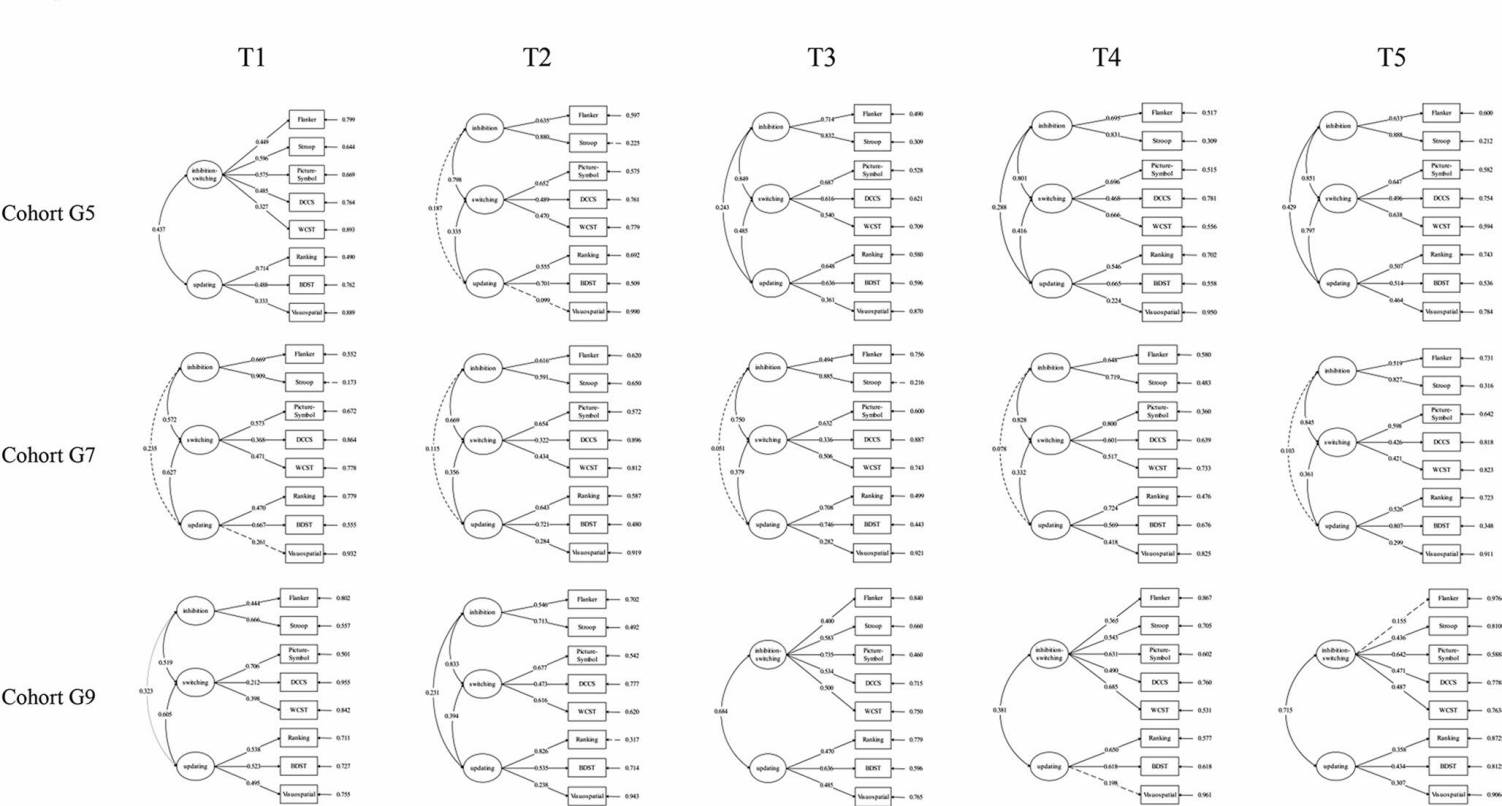
EF component structure across cohorts and waves

The model evaluation fit indices and results of the confirmatory factor analyses are presented in Table S5 and Fig. 1. Generally, the executive function structure exhibited age-dependent dimensionality. At the youngest age (T1 of cohort G5, approximately 10 years old), a two-factor model (M2a: inhibition/switching + updating) provided the best fit. Thereafter, throughout middle adolescence (T2–T5 of cohort G5, T1–T5 of cohort G7, and T1-T2 of cohort G9), a standard three-factor model featuring distinct inhibition, switching, and updating factors was supported. However, in older adolescents (T3 – T5 of cohort G9, 15 + years old), the executive function structure reverted to a two-factor model featuring a combined inhibition/switching factor alongside a separate updating factor (M2a).

Specifically, data from the T1 assessment of the G5 cohort (approximately age 10) indicated that the two-factor model with separate updating and combining inhibition and switching measures (M2a) demonstrated the best fit among the two-factor models. Compared with the M2a, M1 showed poorer fit; the three-factor model (M3) did not fit better than M2a. The two-factor (M2a) model showed that all measures loaded significantly onto their respective latent variables. The two latent factors, updating and the combined inhibition–switching factor, were significantly correlated (*r* = .44). Adolescents with larger updating capacity exhibited higher accuracy in the inhibition–switching conditions. In other words, adolescents with greater updating capacity were less affected by inhibition–switching demands.

Findings from T2 of G5 to T2 of G9 cohort (age: 11.22–15.21) accepted the three-factor model (M3). The three-factor model (M3) had the best model fit. M3 provided the best overall fit (CFI: 0.954–1.000) and improved significantly over other models (except at T5 of cohort G7 marginal differences observed for a minority of indices compared to M2a). All measures loaded significantly onto their respective latent variables (expect the visuospatial task in T2 of cohort G5 and T1 of cohort G7). The three latent factors, updating, inhibition and switch factor, were significantly correlated (*r*s = 0.23 − 0.43) in models at T3, T4, T5 of cohort G5 and T1, T2 of cohort G9. In the G7 cohort models (T1-T5), switching was significantly correlated with both inhibition (*r*s = 0.57 − 0.85) and updating (*r*s = 0.33 − 0.63). In contrast, the association between updating and inhibition was non-significant (*r*s = 0.05 − 0.24).

For the T3 to T5 assessment of G9 cohort, the two-factor model combining inhibition and switching measures (M2a) demonstrated the best fit, compared to M1, M2b, M2c, and M3. All measures loaded significantly onto their respective latent variables (expect the visuospatial task in T4 of G9 cohort and the Flanker in T5 of G9 cohort). The two latent factors, updating and the combined inhibition–switch factor, were significantly correlated (*r*s = 0.38 − 0.72).

### Measure Equivalence

Given the longitudinal nature of the variables, the study specified a series of longitudinal confirmatory factor model to examine measurement invariance across waves for each cohort (See Table S6). Based on the longitudinal confirmatory factor model, the study sequentially tested configural, loading, and intercept invariance. The results indicated that M3 demonstrated an acceptable level of model fit (see Table S5). Specifically, M3 satisfied loading invariance for the G5 (T2 to T5), G7 (T1 to t5) and G9 (T1 to T2) cohorts. The results of test of measurement invariance over time of the two-factor model of cohort G9 (T3 to T5) indicated that M2a did not satisfy the criteria for configural invariance. Next, to examine measurement invariance of M2a across cohorts, a series of multi-group confirmatory factor analyses for configural, metric, and scalar invariance between G5 and G9 groups were conducted. These results suggest that the construct satisfied configural invariance across G5 and G9 participants (See Table S7).

### Criterion Validity

After identifying the optimal model at each time point across the three cohorts, the study examined whether executive functions predicted achievement, planning, peer relationships (acceptance/rejection), and internalizing/externalizing symptoms. Results are presented in Table 1.

**Table 1:** Standardized robust maximum likelihood estimates of EF components on academic performance, planning, peer relationship, internalizing and externalizing symptomatology

Cohort	EF components	T1 score	T1 planning	T2 score	T2 planning	T3 score	T3 planning	T4 score	T4 planning	T5 score	T5 planning
G5	inhibition	**0.318** ^**a^	–0.135^a^	–0.242	–0.223	–0.475	0.280	**–0.471** ^*^	0.002	–0.745	0.493
	switching	–	–	**0.588** ^*^	0.285	**0.770** ^*^	–0.344	**0.740** ^**^	–0.049	1.595	–0.952
	updating	**0.346** ^**^	0.014	**0.225** ^*^	0.100	0.215	0.048	**0.427** ^***^	0.013	–0.896	–0.574
G7	inhibition	0.011	0.075	–0.126	0.043	–0.631	0.075	–0.774	–0.062	–0.274	–0.003
	switching	0.195	0.128	**0.504** ^*^	–0.201	**1.035** ^*^	0.007	1.273	0.169	0.459	–0.087
	updating	**0.468** ^**^	–0.282	0.174	–0.094	–0.018	–0.165	0.034	–0.102	–0.266^+^	–0.040
G9	inhibition	0.048	–0.057	–0.213	–0.230	0.058^a^	0.151^a^	^b^	0.000^a^	–0.285^a^	–0.209^a^
	switching	**0.580** ^*^	0.015	**0.681** ^*^	0.406	–	–	–	–	–	–
	updating	0.198	–0.072	0.176	–0.121	0.196	–0.138	^b^	–0.128	0.603	0.185

Cohort	EF components	T1 RE	T1 AC	T2 RE	T2 AC	T3 RE	T3 AC	T4 RE	T4 AC	T5 RE	T5 AC
G5	inhibition	–0.010^a^	0.055^a^	–0.114	–0.398	–0.189	–0.692	–0.235	–0.223	–0.394	–0.219
	switching	–	–	–0.240	**0.551** ^*^	–0.108	**0.919** ^*^	–1.332	**0.448** ^*^	0.209	0.164
	updating	**–0.270** ^*^	0.001	–0.095	0.102	–0.197	–0.019	–0.329	0.084	–0.370	0.513
G7	inhibition	–0.056	0.031	0.015	–0.341	0.241	–0.406	–0.369	–0.223	0.530	0.026
	switching	–0.031	0.037	–0.265	0.362	**–0.518** ^*^	0.427	0.129	0.094	–0.747	–0.090
	updating	**–0.443** ^*^	0.077	–0.059	–0.079	0.077	–0.187	–0.069	0.053	0.111	0.101
G9	inhibition	–0.452^+ a^	0.138	–0.091	–0.557	–0.343^+ a^	0.151^a^	–0.226^+ a^	–.039^a^	0.069^a^	–0.182^a^
	switching	0.018	–0.167	–0.049	0.672	–	–	–	–	–	–
	updating	–0.033	0.215	**–0.264** ^*^	0.003	0.073	–0.272	0.119	–0.036	–0.169	0.224

Cohort	EF components	T1 INT	T1 EXT	T2 INT	T2 EXT	T3 INT	T3 EXT	T4 INT	T4 EXT	T5 INT	T5 EXT
G5	inhibition	0.086^a^	.022^a^	0.182	–0.076	–0.046	0.069	–0.125	0.046	0.885	1.095
	switching	–	–	–0.149	0.023	–0.050	–0.397	0.140	–0.185	–1.491	–1.978
	updating	–0.172	–0.194	–0.053	–0.093	–0.192	–0.142	**–0.202** ^*^	–0.121	0.741	1.032
G7	inhibition	–0.074	0.000	**–0.370** ^**^	**–0.520** ^***^	–0.439	–0.411	–0.478	–0.774	0.206	0.191
	switching	–0.029	0.037	0.227	**0.398** ^*^	0.438	0.165	0.449	0.611	–0.195	–0.351
	updating	0.159	0.189	–0.116	–0.175	–0.146	–0.045	–0.006	–0.108	0.035	–0.002
G9	inhibition	0.157	0.133	–0.102	–0.383	–0.121^a^	–0.286^a^	–0.123^a^	–0.178^a^	–0.564^a^	–0.417^a^
	switching	–0.266	**–0.453** ^*^	0.031	0.124	–	–	–	–	–	–
	updating	0.124	0.181	0.076	–0.033	0.256	0.223	0.122	0.138	0.767	0.439

+*p* < .10; *p < .05; **p < .01; ***p < .001. Score = achievement performance; RE= peer rejection; AC = peer acceptance; INT = internalizing; EXT = externalizing. Bold denotes significant results

^a^ Standardized robust maximum likelihood estimates of inhibition-switching on criterion variables. ^b^ The model failed to converge

The results generally confirmed the prediction of executive functions on these criterion variables. Executive functions showed significantly positive associations with academic achievement (except the negative association with inhibition at T4 for cohort G5): G5 for switching (T2-T4), inhibition-switching (T1), and updating (T1, T2, T4); G7 for switching (T2, T3) and updating (T1); and G9 for switching (T1-T2). Executive functions were positively associated with peer acceptance, and negatively associated with peer rejection: G5: switching enhanced peer acceptance (T2-T4), while updating reduced rejection (T1), G7: switching (T3) and updating (T1) both decreased peer rejection, and G9: updating negatively predicted rejection (T2). Inhibition showed no significant associations with peer relationships across all waves and cohorts. Executive functions generally decreased symptom outcomes (except the positive prediction of switching on externalizing symptoms at T2 for cohort G7): G5: updating reduced internalizing symptoms (T4); G7: inhibition decreased both internalizing and externalizing symptoms (T2); G9: switching reduced externalizing symptoms (T1). There was no significant prediction of the executive function components on performance of planning measured by Tower of Hanoi (HOT) task.

### Sensitivity Analysis

In addition to confirmatory factor analyses, the study conducted exploratory factor analyses at each cohort and wave as a robustness check. Exploratory factor analyses were performed using principal axis factoring with oblique rotation, allowing cross-loadings among tasks. The results of exploratory factor analyses were consistent with findings from the confirmatory factor analyses (see Table S8). Some factor loadings in certain models were below 0.30 (G5: Visuospatial Memory task at T1 and Dimension Change Card Sort task at T3; G7: Picture Symbol task at T1, Visuospatial Memory task at T1, and Picture Symbol task at T3; G9: Flanker task at T5 and Visuospatial Memory task at T5). Exploratory factor analyses generally supported a two-factor solution in the youngest (around age 10) and oldest (15+) groups and a three-factor solution in middle adolescence. Cross-loadings were minimal, and the task clustering pattern aligned with theoretical expectations for inhibition, switching, and updating. These results indicate that the developmental changes in executive function structure observed in CFA were not driven by model constraints.

To evaluate the robustness of findings to different approaches of handling missing data, the study conducted sensitivity analyses using multiple imputation. Specifically, missing data were handled using multiple imputation with the *mice* package in R (van Buuren & Groothuis-Oudshoorn, 2011). For continuous task variables (e.g., executive function indicators across T1–T5), the study applied predictive mean matching (PMM) as the imputation method. Thirty imputed datasets (m = 30) were generated using 20 iterations for each imputation chain, with a fixed random seed to ensure reproducibility. Across these sensitivity checks, the model fit indices and factor loading patterns were highly similar to those obtained under FIML, and the developmental patterns of executive function structure remained consistent. These results support the robustness of the reported findings despite sample attrition (see Table S9 and Fig. S3).

## Discussion

Executive functioning is an important developing self-regulatory process that has implications for academic, social, and emotional outcomes. Most work in executive function has focused on childhood, and less has examined the development of executive function from early to late adolescence. To address these gaps, the current study investigated the developmental changes in executive function component structure among Chinese adolescents aged 10 to 18 years using a cohort-sequential design. The present study provides novel insights into the developmental organization of executive function components of adolescents in a large, longitudinally followed Chinese sample. Consistent with prior findings (e.g., Karr et al., 2022; Lee et al., 2013), the results reveal a general trend of differentiation of executive function components during early to middle adolescence, followed by a structural re-integration in later adolescence. Specifically, beginning from age 11, executive function tasks clearly segregated into three components (inhibition, shifting, and updating), which persisted until around age 15. However, from that point onward, the structure reorganized into a two-factor model, with inhibition and shifting merging into a single latent factor. This developmental trajectory suggests both differentiation and subsequent reorganization in executive function organization during adolescence.

### Factor Structure

Age-related changes in inhibition, shifting, and updating capacities showed significant task-specific differences. Compared to inhibition and updating, the study observed a rapid increase in shifting with age. This result supports previous theoretical framework and studies (Best & Miller, 2010; Lee et al., 2013). One possible explanation is that the development stages and trajectories of the three components are not consistent (Best & Miller, 2010; Folker et al., 2025). Specifically, inhibition develops rapidly during the preschool years, followed by more modest, linear improvements through adolescence; updating follows a linear trajectory from preschool through adolescence; the ability to shift improves with age. Moreover, cross-cultural research suggested accelerated executive function development trajectories in Chinese children compared to their Western counterparts (e.g., Xu et al., 2020), which may lead to earlier attainment of adult-level executive functioning maturity during adolescence. As shown in current study result, both the differentiation of executive function components and their developmental progression occur at earlier ages in Chinese adolescents relative to their Western peers.

Although age-related differences in inhibition, shifting, and updating showed some similarities in trajectories, they do not provide a direct test of the relations among the measures. A unique contribution of this study is that it shows how the structure of executive function varies over the second decade of life. The findings revealed a development in executive function structure that reflects both differentiation and functional reorganization across adolescence. Specifically, adolescents aged 10–11 showed a two-factor structure in which inhibition and switching tasks loaded onto a common latent construct, while updating formed a separate factor. By ages 11–15, this structure transitioned into a three-factor model with clearly separable inhibition, switching, and updating components. However, from age 15 onward, the data again favored a two-factor solution wherein inhibition and switching merged. This dynamic pattern of factor structure change suggests that executive functions are not organized in a static fashion across development, but rather undergo systematic shifts in response to neurocognitive maturation (Diamond, 2013; Zelazo, 2020).

During early adolescence, the overlap between inhibition and switching likely reflects shared reliance on interference control processes and immature prefrontal circuitry. This finding aligns with the interactive specialization account (Johnson & Munakata, 2005), which emphasizes that developing systems initially depend on overlapping neural networks that later differentiate with experience and increasing cognitive demands. The emergence of a three-factor structure in middle adolescence coincides with a period of marked functional specialization, supported by the progressive maturation of prefrontal and parietal brain regions (Fiske & Holmboe, 2019). The increasing separability of inhibition, switching, and updating during this stage enables adolescents to apply more targeted executive strategies in academic, social, and emotional contexts.

Findings from the current study stand in similarities and differences to extant studies. The findings are similar to Lee et al.’s (2013) in which they found separation between updating and an inhibition-switching factor across 6 to 14 years, and that differentiation into a three-factor structure occurs over a protracted period, with signs of early differentiation emerging at age 11 and reaching some stability at age 15. About the time of switching differentiation from inhibition, Monette et al. (2015) inferred around 9 or 10 years old. A possible reason for this difference is that Lee et al.’s (2013) measured inhibition and switching using different metrics (correct number and reaction time) within the same task. The same task environment would obscure distinctions between inhibition and switching due methodological overlap, delaying their differentiation; although Lee et al. controlled for the effect of metrics by regression, the ability to characterize switching by reaction time would be affected by reaction speed. By nature, both inhibition and shifting require a ‘stop’ process to some degree (though shifting also requires a ‘change’ as well, i.e. the participant has to change from the prepotent response, rather than simply inhibit any response), so it could be that some interference control processes are shared between these two processes (Friedman & Miyake, 2004).

Unusually, the results of the study also revealed a stable two-factor structure at higher grades (T3 to T5 of cohort G9) rather than a stable three-factor structure. It was also found that the two-factor model (M2a) at G9 (T3 - T5) failed to meet configurational invariance. This might be due to the fact that the Flanker task was too simple for late adolescents, and the results were affected by the ceiling effect (Lee et al., 2013; Zelazo et al., 2020). Multi-group confirmatory factor analyses further revealed configural invariance between G5 and G9, supporting comparable factor structures across cohorts. The findings further corroborate the dynamic nature of cognitive ability differentiation and reorganization during childhood and adolescence (Dreisbach et al., 2024; Egner, 2023; Hommel et al., 2024), while revealing a reciprocal relationship between inhibition and shifting (Karr et al., 2022).

The return to a two-factor structure in late adolescence should not be interpreted as a regression, as performance on all tasks continued to improve across time points, which was unlike the dedifferentiation seen in older adulthood (Karr et al., 2022). Rather, the merging of inhibition and switching may represent a functional reintegration of cognitive stability and flexibility mechanisms. According to the meta-control framework (Dreisbach et al., 2024), mature executive control depends not on the independence of inhibition and switching but on their coordination within a unified, adaptive control system. From this perspective, the merging of inhibition and switching in older adolescents reflects the integration of goal maintenance and cognitive flexibility functions, which together support efficient regulation in increasingly complex real-world environments. Taken together, these results indicate that differentiation and integration are not opposing outcomes, but instead represent complementary phases in the development of cognitive control. The transition from undifferentiated control to specialized components, followed by strategic reintegration, reflects an adaptive developmental sequence in the organization of executive function.

### Criterion Validity

The predictive models are intentionally broad, encompassing multiple executive function components, outcomes, and developmental periods (cohorts, and waves). This scope increases the risk of Type I error, as no formal multiple-comparison correction was applied. To address this, the study focused on identifying and interpreting the most consistent effects across cohorts and time, while explicitly labeling isolated findings as exploratory. This strategy allows the results to inform future hypothesis generation while minimizing the risk of overstating single-wave or single-cohort effects.

The study revealed relevant findings regarding the predictive validity of executive function components. Switching and updating abilities predicted academic achievement, underscoring their fundamental role in learning processes (Ahmed et al., 2019). These two components may facilitate flexible thinking, goal monitoring, and the integration of new information, which are essential for effective academic performance. In contrast, inhibition showed weaker and less stable associations with academic outcomes. This discrepancy may reflect culturally specific developmental trajectories of self-regulatory processes (Kitayama & Salvador, 2024), particularly in collectivistic contexts such as East Asia, where inhibitory control is often emphasized early in life through parental and educational practices. Such early consolidation may lead to reduced variability in inhibition among adolescents, thereby weakening its predictive power for academic differences.

With regard to peer relationships, switching ability emerged as a significant predictor of peer acceptance, likely due to its role in enabling adolescents to adapt their behavior across diverse social settings and respond flexibly to interpersonal demands (Zelazo, 2020). Updating capacity was negatively associated with peer rejection, suggesting that strong working memory helps individuals to process social cues accurately, regulate emotional reactions, and maintain coherent social narratives during interactions. These findings indicate that different components may support distinct aspects of social functioning, and their relevance extends beyond cognitive domains into the social-emotional sphere. Collectively, these patterns validate the structural distinctions among executive function components and highlight their practical significance for adolescent development within both academic and interpersonal domains (Wang & Zhou, 2019).

Compared with other outcomes, executive function components showed relatively weaker predictive effects on internalizing and externalizing problem behaviors. This finding contrasts with some prior research that has established robust links between executive dysfunction and behavioral or emotional difficulties (Yang et al., 2022; Zelazo, 2020). One possible explanation is that executive function, rather than serving as a robust direct predictor of problem outcomes, operates more as a moderator, for example, between sensation seeking and substance use (Kim-Spoon et al., 2016). Moreover, cultural norms regarding emotional expression and behavioral control may attenuate the observable effects of executive function on these outcomes. For instance, in societies where emotional restraint is encouraged, internalizing and externalizing symptoms may manifest in less overt ways, complicating their association with cognitive control measures (Hassan & Schmidt, 2022). Thus, while executive function plays a crucial role in cognitive and social development, its influence on mental health outcomes may be more complex and warrants further investigation using integrative models.

### Limitations

Several limitations should be acknowledged when interpreting these results. First, a persistent challenge in EF research is the “task impurity” problem, whereby even well-established tasks capture overlapping nonexecutive cognitive processes. To mitigate this concern, this study included multiple measures per executive function component and employed confirmatory factor modelling to separate task-specific variance from the specified latent factors (i.e., executive function components). However, the issue cannot be fully eliminated. The interpretation of method factors for executive function tasks is less straightforward than for questionnaires, because task-specific variance reflects a mixture of perceptual, motor, and other non-executive processes (Willoughby et al., 2012). Advanced strategies in modeling task-specific variance or adopting multitrait–multimethod approaches may provide a more refined separation of executive function constructs, and these approaches should be considered in future research. Second, the study experienced significant missing in older cohorts, particularly during school transitions, which may have introduced bias in the late adolescent findings. Third, the current study could not definitively determine whether the observed reorganization reflects neurodevelopmental changes, environmental demands, or their interaction. Multimethod approaches incorporating neuroimaging, behavioral experimental and environmental measures methods could help elucidate these underlying mechanisms in future research. Finally, measurement modality may have contributed to the observed differences between updating and inhibition/shifting abilities. Specifically, two tasks for updating (i.e., the Backwards Digit Span task and 2-List List sorting task) were assessed using one-on-one individual tasks, while tasks for inhibition and shifting, and one task for updating (i.e., Visuospatial Memory task) were measured in group settings. This variation in administration format could have influenced performance, as individual assessments often allow for more tailored instruction and reduced distractions, potentially enhancing performance compared to group-based testing (Bignardi et al., 2021). The findings in this study should be verified in future research employing the same administration mode (individual or group) for measuring executive function.

A further limitation involves the potential influence of practice effects, as participants completed the same set of executive function tasks across multiple waves. While the observed developmental patterns align with prior longitudinal studies (Friedman & Miyake, 2017; Karr et al., 2022), repeated exposure may nevertheless have contributed to performance improvements through increased familiarity with task procedures. Future research could mitigate this concern by using parallel task versions, introducing longer retest intervals, or statistically modeling practice-related variance to better isolate developmental change.

## Conclusion

Few studies have systematically examined the development of EF structure during the second decade of life, a critical period marked by significant neurofunctional maturation and profound environmental transitions. The present study charts a developmental sequence in the organization of executive function from early to late adolescence, revealing a pattern that unfolds in distinct phases. Around age ten, executive functioning is characterized by an integrated inhibition–shifting factor and updating factor. This integrated profile gives way in early to middle adolescence to a fully differentiated structure in which inhibition, shifting, and updating operate as three separable components. By the late adolescent years, however, inhibition and shifting converge once again into a unified factor, while updating remains independent. The study also shows that the functional relevance of executive components varies developmentally, with updating and shifting playing key roles in academic performance and social adjustment. These associations underscore the need to consider age-specific configurations of executive functioning when examining its links to real-world outcomes. Taken together, the findings demonstrate that the development of executive function across adolescence is marked by alternating phases of differentiation and integration. Mapping these structural transitions provides a clearer understanding of how cognitive control systems reorganize as individuals move toward adulthood and offers a developmental framework for interpreting age-related variability in executive functioning across the second decade of life.

## Supplementary Information

Below is the link to the electronic supplementary material.

Supplementary Material 1

## Data Availability

Data supporting the findings of this study are available from the corresponding author upon reasonable request.
