# Supplementary material for: Developmental Changes in the Structure of Executive Function from Early To Late Adolescence
Source: J Youth Adolesc. 2025 Dec 4;55(4):982–97. doi: 10.1007/s10964-025-02293-7 (PMC13076501; doi:10.1007/s10964-025-02293-7)

**Online Supplemental Materials for**

**Developmental Changes in the Structure of Executive Function from Early to Late Adolescence**

**Authors’ note:**

We developed these materials to provide additional technical information and to keep the main manuscript from becoming needlessly long.

**Table S1**

*Family Characteristics*

|  | Male | Female |
| --- | --- | --- |
| Mother’s education level |  |  |
| elementary school education or below | 91 (19.2%) | 82 (21.3%) |
| junior high school education | 221 (46.7%) | 171 (44.4%) |
| high school education | 105 (22.2%) | 70 (18.2%) |
| college education and more | 54 (11.4%) | 61 (15.9%) |
| not report | 2 (0.4%) | 1 (0.3%) |
| Father’s education level |  |  |
| elementary school education or below | 47 (9.9%) | 47 (12.2%) |
| junior high school education | 232 (49.0%) | 180 (46.8%) |
| high school education | 124 (26.2%) | 90 (23.4%) |
| college education and more | 66 (14.0%) | 66 (17.1%) |
| not report | 4 (0.8%) | 1 (0.3%) |
| Household monthly income |  |  |
| < ¥3000 | 68 (14.4%) | 69 (17.9%) |
| ¥3000 - ¥5000 | 167 (35.3%) | 133 (34.5%) |
| ¥5000 - ¥7000 | 132 (27.9%) | 97 (25.2%) |
| > ¥7000 | 103 (21.7%) | 85 (22.1%) |
| not report | 3 (0.6%) | 1 (0.3%) |

**Table S2**

*Descriptive Statistics and Correlations*

| **Cohort G5** | | | | | | | | | | |
| --- | --- | --- | --- | --- | --- | --- | --- | --- | --- | --- |
| **T1** | | | | | | | | | | |
|  | Variable | M | SD | 1 | 2 | 3 | 4 | 5 | 6 | 7 |
| 1 | Flanker | 17.96 | 3.15 |  |  |  |  |  |  |  |
| 2 | Stroop | 15.96 | 3.62 | .281^***^ |  |  |  |  |  |  |
| 3 | PS | 0.63 | 0.14 | .255^***^ | .352^***^ |  |  |  |  |  |
| 4 | DCCS | 0.70 | 0.24 | .244^***^ | .269^***^ | .233^***^ |  |  |  |  |
| 5 | WCST | 1.45 | 1.01 | .059 | .154^*^ | .233^***^ | .196^***^ |  |  |  |
| 6 | VM | 4.76 | 1.08 | .067 | .208^***^ | .196^***^ | .149^*^ | .141^*^ |  |  |
| 7 | Rank | 4.36 | 1.20 | .104 | .119 | .109 | .193^**^ | .106 | .230^***^ |  |
| 8 | BDST | 3.98 | 1.08 | .094 | .025 | .089 | .105 | .120 | .097 | .369^***^ |
| **T2** | | | | | | | | | | |
|  | Variable | M | SD | 1 | 2 | 3 | 4 | 5 | 6 | 7 |
| 1 | Flanker | 18.13 | 3.03 |  |  |  |  |  |  |  |
| 2 | Stroop | 15.81 | 4.08 | .502^***^ |  |  |  |  |  |  |
| 3 | PS | 0.67 | 0.16 | .336^***^ | .493^***^ |  |  |  |  |  |
| 4 | DCCS | 0.73 | 0.23 | .291^***^ | .308^***^ | .273^***^ |  |  |  |  |
| 5 | WCST | 1.82 | 1.16 | .187^**^ | .280^***^ | .316^***^ | .287^***^ |  |  |  |
| 6 | VM | 4.86 | 1.09 | .124^*^ | .227^***^ | .216^***^ | .136^*^ | .147^*^ |  |  |
| 7 | Rank | 4.37 | 1.42 | .030 | .056 | .055 | .086 | .127^*^ | .058 |  |
| 8 | BDST | 4.05 | 1.34 | .036 | .132^*^ | .134^*^ | .161^**^ | .111 | .020 | .394^***^ |
| **T3** | | | | | | | | | | |
|  | Variable | M | SD | 1 | 2 | 3 | 4 | 5 | 6 | 7 |
| 1 | Flanker | 17.80 | 3.56 |  |  |  |  |  |  |  |
| 2 | Stroop | 15.88 | 4.15 | .451^***^ |  |  |  |  |  |  |
| 3 | PS | 0.68 | 0.17 | .358^***^ | .409^***^ |  |  |  |  |  |
| 4 | DCCS | 0.78 | 0.21 | .327^***^ | .391^***^ | .392^***^ |  |  |  |  |
| 5 | WCST | 2.10 | 1.23 | .276^***^ | .329^***^ | .406^***^ | .344^***^ |  |  |  |
| 6 | VM | 5.13 | 1.06 | .206^***^ | .109 | .189^**^ | .261^***^ | .109 |  |  |
| 7 | Rank | 4. 81 | 1.24 | .011 | .087 | .207^***^ | .184^**^ | .097 | .231^***^ |  |
| 8 | BDST | 4.37 | 1.15 | .009 | .176^**^ | .232^***^ | .144^*^ | .184^**^ | .196^***^ | .430^***^ |
| **T4** | | | | | | | | | | |
|  | Variable | M | SD | 1 | 2 | 3 | 4 | 5 | 6 | 7 |
| 1 | Flanker | 17.54 | 4.08 |  |  |  |  |  |  |  |
| 2 | Stroop | 15.82 | 4.34 | .528^***^ |  |  |  |  |  |  |
| 3 | PS | 0.70 | 0.18 | .441^***^ | .394^***^ |  |  |  |  |  |
| 4 | DCCS | 0.77 | 0.18 | .231^***^ | .275^***^ | .334^***^ |  |  |  |  |
| 5 | WCST | 2.23 | 1.29 | .332^***^ | .428^***^ | .455^***^ | .322^***^ |  |  |  |
| 6 | VM | 5.23 | 1.18 | .105 | .192^**^ | .232^***^ | .135^*^ | .161^**^ |  |  |
| 7 | Rank | 4.97 | 1.30 | .058 | .109 | .109 | .091 | .119^*^ | .106 |  |
| 8 | BDST | 4.40 | 1.24 | .120^*^ | .117 | .175^**^ | .060 | .233^***^ | .101 | .380^***^ |
| **T5** | | | | | | | | | | |
|  | Variable | M | SD | 1 | 2 | 3 | 4 | 5 | 6 | 7 |
| 1 | Flanker | 18.05 | 3.57 |  |  |  |  |  |  |  |
| 2 | Stroop | 17.12 | 3.40 | .469^***^ |  |  |  |  |  |  |
| 3 | PS | 0.72 | 0.19 | .333^***^ | .441^***^ |  |  |  |  |  |
| 4 | DCCS | 0.82 | 0.19 | .295^***^ | .341^***^ | .281^***^ |  |  |  |  |
| 5 | WCST | 2.73 | 1.26 | .321^***^ | .386^***^ | .426^***^ | .336^***^ |  |  |  |
| 6 | VM | 5.43 | 1.09 | .155^*^ | .172^**^ | .261^***^ | .177^**^ | .301^***^ |  |  |
| 7 | Rank | 5.22 | 1.21 | .084 | .171^**^ | .275^***^ | .146^*^ | .243^***^ | .203^***^ |  |
| 8 | BDST | 4.63 | 1.19 | .083 | .197^**^ | .309^***^ | .062 | .266^***^ | .209^***^ | .313^***^ |
| **Cohort G7** | | | | | | | | | | |
| **T1** | | | | | | | | | | |
|  | Variable | M | SD | 1 | 2 | 3 | 4 | 5 | 6 | 7 |
| 1 | Flanker | 19.12 | 1.76 |  |  |  |  |  |  |  |
| 2 | Stroop | 17.33 | 3.17 | .585^***^ |  |  |  |  |  |  |
| 3 | PS | 0.69 | 0.17 | .223^*^ | .366^***^ |  |  |  |  |  |
| 4 | DCCS | 0.76 | 0.22 | .260^**^ | .083 | .153 |  |  |  |  |
| 5 | WCST | 2.02 | 1.14 | .106 | .130 | .278^**^ | .198^*^ |  |  |  |
| 6 | VM | 5.09 | 1.17 | .138 | .021 | .154 | .139 | .147 |  |  |
| 7 | Rank | 4.84 | 1.32 | .017 | .060 | .112 | .075 | .102 | .142 |  |
| 8 | BDST | 4.34 | 1.11 | .062 | .128 | .180^*^ | .208^*^ | .234^**^ | .105 | .330^***^ |
| **T2** | | | | | | | | | | |
|  | Variable | M | SD | 1 | 2 | 3 | 4 | 5 | 6 | 7 |
| 1 | Flanker | 18.81 | 2.61 |  |  |  |  |  |  |  |
| 2 | Stroop | 17.15 | 3.26 | .199^**^ |  |  |  |  |  |  |
| 3 | PS | 0.72 | 0.15 | .247^***^ | .236^***^ |  |  |  |  |  |
| 4 | DCCS | 0.77 | 0.24 | .158^*^ | .081 | .229^***^ |  |  |  |  |
| 5 | WCST | 2.30 | 1.17 | .097 | .232^***^ | .283^***^ | .099 |  |  |  |
| 6 | VM | 5.09 | 1.26 | .108 | .053 | .220^***^ | .118 | .020 |  |  |
| 7 | Rank | 4.77 | 1.41 | .082 | .025 | .100 | .081 | .103 | .172^**^ |  |
| 8 | BDST | 4.03 | 1.18 | .113 | .110 | .145^*^ | .110 | .127 | .180^**^ | .469^***^ |
| **T3** | | | | | | | | | | |
|  | Variable | M | SD | 1 | 2 | 3 | 4 | 5 | 6 | 7 |
| 1 | Flanker | 18.60 | 2.74 |  |  |  |  |  |  |  |
| 2 | Stroop | 16.78 | 3.90 | .460^***^ |  |  |  |  |  |  |
| 3 | PS | 0.73 | 0.17 | .260^***^ | .429^***^ |  |  |  |  |  |
| 4 | DCCS | 0.80 | 0.19 | .097 | .202^**^ | .148^*^ |  |  |  |  |
| 5 | WCST | 2.32 | 1.19 | .172^***^ | .323^***^ | .309^***^ | .302^***^ |  |  |  |
| 6 | VM | 5.39 | 1.10 | .036 | .055 | .202^**^ | .125^*^ | .149^*^ |  |  |
| 7 | Rank | 5.00 | 1.39 | .009 | .034 | .146^*^ | .147^*^ | .078 | .169^**^ |  |
| 8 | BDST | 4.48 | 1.34 | .029 | .041 | .186^**^ | .107 | .098 | .185^**^ | .534^***^ |
| **T4** | | | | | | | | | | |
|  | Variable | M | SD | 1 | 2 | 3 | 4 | 5 | 6 | 7 |
| 1 | Flanker | 18.64 | 3.08 |  |  |  |  |  |  |  |
| 2 | Stroop | 17.39 | 2.87 | .391^***^ |  |  |  |  |  |  |
| 3 | PS | 0.77 | 0.18 | .426^***^ | .411^***^ |  |  |  |  |  |
| 4 | DCCS | 0.81 | 0.21 | .409^***^ | .297^***^ | .461^***^ |  |  |  |  |
| 5 | WCST | 2.51 | 1.22 | .215^**^ | .237^***^ | .425^***^ | .354^***^ |  |  |  |
| 6 | VM | 5.46 | 1.26 | .197^**^ | .205^**^ | .188^**^ | .262^***^ | .180^*^ |  |  |
| 7 | Rank | 5.21 | 1.25 | .026 | .050 | .200^**^ | .112 | .084 | .293^***^ |  |
| 8 | BDST | 4.59 | 1.27 | –.077 | .014 | .126 | .006 | .071 | .230^***^ | .419^***^ |
| **T5** | | | | | | | | | | |
|  | Variable | M | SD | 1 | 2 | 3 | 4 | 5 | 6 | 7 |
| 1 | Flanker | 18.96 | 2.68 |  |  |  |  |  |  |  |
| 2 | Stroop | 17.81 | 2.81 | .365^***^ |  |  |  |  |  |  |
| 3 | PS | 0.80 | 0.14 | .323^***^ | .394^***^ |  |  |  |  |  |
| 4 | DCCS | 0.83 | 0.19 | .249^***^ | .264^***^ | .230^***^ |  |  |  |  |
| 5 | WCST | 2.96 | 1.20 | .194^***^ | .258^***^ | .252^***^ | .232^***^ |  |  |  |
| 6 | VM | 5.47 | 1.13 | .064 | .145^*^ | .104 | .058 | .013 |  |  |
| 7 | Rank | 5.29 | 1.28 | .026 | .059 | .168^**^ | .035 | .078 | .150^*^ |  |
| 8 | BDST | 4.71 | 1.20 | .034 | .055 | .188^**^ | .081 | .129^*^ | .248^***^ | .424^***^ |
| **Cohort G9** | | | | | | | | | | |
| **T1** | | | | | | | | | | |
|  | Variable | M | SD | 1 | 2 | 3 | 4 | 5 | 6 | 7 |
| 1 | Flanker | 19.30 | 1.88 |  |  |  |  |  |  |  |
| 2 | Stroop | 18.46 | 1.78 | .297^***^ |  |  |  |  |  |  |
| 3 | PS | 0.75 | 0.14 | .159^*^ | .262^***^ |  |  |  |  |  |
| 4 | DCCS | 0.80 | 0.22 | .157^*^ | .051 | .101 |  |  |  |  |
| 5 | WCST | 2.58 | 1.24 | .067 | .092 | .290^***^ | .177^**^ |  |  |  |
| 6 | VM | 5.21 | 1.15 | .138^*^ | .107 | .248^***^ | .037 | .083 |  |  |
| 7 | Rank | 4.74 | 1.24 | .018 | .125^*^ | .225^***^ | .114 | .045 | .273^***^ |  |
| 8 | BDST | 4.20 | 1.03 | .055 | .121 | .213^***^ | .094 | .202^***^ | .235^***^ | .294^***^ |
| **T2** | | | | | | | | | | |
|  | Variable | M | SD | 1 | 2 | 3 | 4 | 5 | 6 | 7 |
| 1 | Flanker | 18.97 | 2.66 |  |  |  |  |  |  |  |
| 2 | Stroop | 17.81 | 2.64 | .336^***^ |  |  |  |  |  |  |
| 3 | PS | 0.78 | 0.15 | .324^***^ | .394^***^ |  |  |  |  |  |
| 4 | DCCS | 0.85 | 0.20 | .180^**^ | .218^***^ | .308^***^ |  |  |  |  |
| 5 | WCST | 2.62 | 1.25 | .274^***^ | .352^***^ | .412^***^ | .312^***^ |  |  |  |
| 6 | VM | 5.08 | 1.11 | .022 | .017 | .170^**^ | .120 | .090 |  |  |
| 7 | Rank | 4.60 | 1.32 | .082 | .180^*^ | .200^**^ | .232^**^ | .162^*^ | .219^**^ |  |
| 8 | BDST | 4.14 | 1.11 | .071 | .106 | .106 | .150^*^ | .234^**^ | .038 | .450^***^ |
| **T3** | | | | | | | | | | |
|  | Variable | M | SD | 1 | 2 | 3 | 4 | 5 | 6 | 7 |
| 1 | Flanker | 19.43 | 1.90 |  |  |  |  |  |  |  |
| 2 | Stroop | 18.39 | 2.28 | .089 |  |  |  |  |  |  |
| 3 | PS | 0.83 | 0.13 | .159^*^ | .451^***^ |  |  |  |  |  |
| 4 | DCCS | 0.86 | 0.18 | .120 | .376^***^ | .337^***^ |  |  |  |  |
| 5 | WCST | 3.00 | 1.22 | .292^***^ | .188^*^ | .374^***^ | .293^***^ |  |  |  |
| 6 | VM | 5.48 | 1.16 | .258^***^ | .070 | .246^***^ | .279^***^ | .070 |  |  |
| 7 | Rank | 5.08 | 1.18 | .129 | .076 | .246^***^ | .214^**^ | .105 | .248^***^ |  |
| 8 | BDST | 4.55 | 1.11 | .164^*^ | .208^**^ | .360^***^ | .285^***^ | .175^*^ | .278^***^ | .303^***^ |
| **T4** | | | | | | | | | | |
|  | Variable | M | SD | 1 | 2 | 3 | 4 | 5 | 6 | 7 |
| 1 | Flanker | 19.63 | 0.87 |  |  |  |  |  |  |  |
| 2 | Stroop | 18.68 | 1.81 | .363^***^ |  |  |  |  |  |  |
| 3 | PS | 0.85 | 0.13 | .250^***^ | .308^***^ |  |  |  |  |  |
| 4 | DCCS | 0.84 | 0.22 | .051 | .260^***^ | .304^***^ |  |  |  |  |
| 5 | WCST | 3.19 | 1.19 | .239^**^ | .380^***^ | .417^***^ | .325^**^ |  |  |  |
| 6 | VM | 5.63 | 1.13 | .129 | .141 | .090 | .110 | .177^*^ |  |  |
| 7 | Rank | 5.24 | 1.18 | .079 | .083 | .146 | .177^*^ | .138 | .156^*^ |  |
| 8 | BDST | 4.62 | 1.25 | .059 | .130 | .150 | .102 | .192^*^ | .068 | .405^***^ |
| **T5** | | | | | | | | | | |
|  | Variable | M | SD | 1 | 2 | 3 | 4 | 5 | 6 | 7 |
| 1 | Flanker | 19.43 | 1.68 |  |  |  |  |  |  |  |
| 2 | Stroop | 18.57 | 1.88 | .080 |  |  |  |  |  |  |
| 3 | PS | 0.87 | 0.12 | .043 | .304^***^ |  |  |  |  |  |
| 4 | DCCS | 0.89 | 0.19 | .100 | .200^*^ | .277^***^ |  |  |  |  |
| 5 | WCST | 3.53 | 1.12 | .082 | .180^*^ | .291^***^ | .317^***^ |  |  |  |
| 6 | VM | 5.53 | 1.35 | .063 | .205^*^ | .172^*^ | .116 | .084 |  |  |
| 7 | Rank | 5.35 | 1.23 | .006 | .027 | .215^**^ | .074 | .131 | .085 |  |
| 8 | BDST | 4.83 | 1.15 | .171^*^ | .133 | .217^**^ | .072 | .143 | .091 | .208^**^ |

*Note.* Flanker = Flanker task; Stroop = Stroop task; PS = Picture Symbol task; DCCS = Dimension Change Card Sort task; WCST = Wisconsin Card Sorting task; VM = Visuospatial Memory task; Rank = 2-List List Sorting; BDST = Backwards Digit Span task.^*^*p* < .05; ^**^*p* < .01; ^***^*p* < .001.

**Table S3**

*Results of repeated measures analysis of variance*

| **Source** | ***df*** | ***F*-value** | ***p*-value** | **η²p** | **Post-hoc Comparisons (if significant)** |
| --- | --- | --- | --- | --- | --- |
| **Flanker** |  |  |  |  |  |
| **Within-Subjects** |  |  |  |  |  |
| Wave | 4, 1688 | 2.31 | .056 | .01 |  |
| Wave × Cohort | 8, 1688 | 1.06 | .385 | .01 |  |
| Wave × Gender | 4, 1688 | 3.26 | .011 | .01 |  |
| Wave × Cohort × Gender | 8, 1688 | 1.57 | .129 | .01 |  |
| **Between-Subjects** |  |  |  |  |  |
| Cohort | 2, 422 | 23.49 | < .001 | .10 | G5 < G7/G9 |
| Gender | 1, 422 | 7.51 | .006 | .02 | boy < girl |
| Cohort × Gender | 2, 422 | 1.11 | .330 | .01 |  |
|  |  |  |  |  |  |
| **Stroop** |  |  |  |  |  |
| **Within-Subjects** |  |  |  |  |  |
| Wave | 4, 1468 | 3.01 | .016 | .01 | T3/T4 < T5 |
| Wave × Cohort | 8. 1468 | 3.38 | < .001 | .02 |  |
| Wave × Gender | 4, 1468 | 2.80 | .025 | .01 |  |
| Wave × Cohort × Gender | 8, 1468 | 0.99 | .444 | .01 |  |
| **Between-Subjects** |  |  |  |  |  |
| Cohort | 2, 367 | 35.44 | < .001 | .16 | G5 < G7 < G9 |
| Gender | 1, 367 | 5.66 | .018 | .02 | boy < girl |
| Cohort × Gender | 2, 367 | 1.79 | .168 | .01 |  |
|  |  |  |  |  |  |
| **PS** |  |  |  |  |  |
| **Within-Subjects** |  |  |  |  |  |
| Wave | 4, 1736 | 30.00 | < .001 | .07 | T1 < T2 <T3/T4/T5 |
| Wave × Cohort | 8, 1736 | 1.04 | .415 | .01 |  |
| Wave × Gender | 4, 1736 | 0.91 | .459 | .002 |  |
| Wave × Cohort × Gender | 8, 1736 | 1.39 | .197 | .01 |  |
| **Between-Subjects** |  |  |  |  |  |
| Cohort | 2, 434 | 67.65 | < .001 | .24 | G5 < G7 < G9 |
| Gender | 1, 434 | 9.95 | .002 | .02 | boy < girl |
| Cohort × Gender | 2, 434 | 1.61 | .201 | .01 |  |
|  |  |  |  |  |  |
| **DCCS** |  |  |  |  |  |
| **Within-Subjects** |  |  |  |  |  |
| Wave | 4, 1696 | 8.01 | < .001 | .02 | T1 < T2, T1 < T3, T1 < T5, T4 < T5 |
| Wave × Cohort | 8, 1696 | 2.55 | .009 | .01 |  |
| Wave × Gender | 4, 1696 | 0.49 | .747 | .001 |  |
| Wave × Cohort × Gender | 8, 1696 | 0.70 | .693 | .003 |  |
| **Between-Subjects** |  |  |  |  |  |
| Cohort | 2, 424 | 29.93 | < .001 | .12 | G5/G7 < G9 |
| Gender | 1, 424 | 1.91 | .168 | .004 | boy < girl |
| Cohort × Gender | 2, 424 | 2.98 | .052 | .014 |  |
|  |  |  |  |  |  |
| **WCST** |  |  |  |  |  |
| **Within-Subjects** |  |  |  |  |  |
| Wave | 4, 1652 | 44.45 | < .001 | .10 | T1/T2 < T3/T4 < T5, |
| Wave × Cohort | 8, 1652 | 2.71 | .006 | .01 |  |
| Wave × Gender | 4, 1652 | 1.39 | .236 | .003 |  |
| Wave × Cohort × Gender | 8, 1652 | 0.69 | .705 | .003 |  |
| **Between-Subjects** |  |  |  |  |  |
| Cohort | 2, 413 | 66.94 | < .001 | .25 | G5 < G7 < G9 |
| Gender | 1, 413 | 23.86 | < .001 | .06 | boy < girl |
| Cohort × Gender | 2, 413 | 2.90 | .056 | .01 |  |
|  |  |  |  |  |  |
| **VM** |  |  |  |  |  |
| **Within-Subjects** |  |  |  |  |  |
| Wave | 4, 1700 | 12.42 | < .001 | .03 | T1/T2 < T3/T4/T5, |
| Wave × Cohort | 8, 1700 | 1.85 | .065 | .01 |  |
| Wave × Gender | 4, 1700 | 0.41 | .800 | .001 |  |
| Wave × Cohort × Gender | 8, 1700 | 3.03 | .002 | .01 |  |
| **Between-Subjects** |  |  |  |  |  |
| Cohort | 2, 425 | 14.54 | < .001 | .06 | G5 < G7/G9 |
| Gender | 1, 425 | 7.00 | .008 | .02 | boy > girl |
| Cohort × Gender | 2, 425 | 7.60 | < .001 | .04 |  |
|  |  |  |  |  |  |
| **Rank** |  |  |  |  |  |
| **Within-Subjects** |  |  |  |  |  |
| Wave | 4, 1456 | 15.42 | < .001 | .04 | T1/T2 < T4/T5, T3 < T5 |
| Wave × Cohort | 8, 1456 | 1.84 | .066 | .01 |  |
| Wave × Gender | 4, 1456 | 1.50 | .200 | .004 |  |
| Wave × Cohort × Gender | 8, 1456 | 1.02 | .421 | .006 |  |
| **Between-Subjects** |  |  |  |  |  |
| Cohort | 2, 364 | 9.32 | < .001 | .05 | G5 < G7/G9 |
| Gender | 1, 364 | 1.50 | .222 | .004 | boy > girl |
| Cohort × Gender | 2, 364 | 0.17 | .846 | .001 |  |
|  |  |  |  |  |  |
| **BDST** |  |  |  |  |  |
| **Within-Subjects** |  |  |  |  |  |
| Wave | 4, 1460 | 19.11 | < .001 | .05 | T1/T2 < T3 < T5, T2 < T4 < T5 |
| Wave × Cohort | 8, 1460 | 0.88 | .529 | .01 |  |
| Wave × Gender | 4, 1460 | 0.11 | .980 | .000 |  |
| Wave × Cohort × Gender | 8, 1460 | 1.68 | .100 | .01 |  |
| **Between-Subjects** |  |  |  |  |  |
| Cohort | 2, 365 | 5.04 | .007 | .03 | G5 < G7/G9 |
| Gender | 1, 365 | 0.01 | .931 | .000 |  |
| Cohort × Gender | 2, 365 | 1.06 | .349 | .01 |  |

**Table S4**

*Comparison of Performance on Individually and Group-Administered EF Tasks: Descriptive Statistics and Paired-Sample t Test Results*

|  |  | group | individual | *t* | *p* |
| --- | --- | --- | --- | --- | --- |
| G7 | T1 | 0.006 ± 0.562 | 0.000 ± 0.827 | 0.115 | 0.454 |
|  | T2 | –0.027 ± 0.646 | 0.004 ± 0.837 | –0.522 | 0.301 |
|  | T3 | –0.002 ± 0.67 | 0.002 ± 0.846 | –0.061 | 0.476 |
|  | T4 | –0.010 ± 0.664 | 0.000 ± 0.831 | –0.170 | 0.433 |
|  | T5 | –0.010 ± 0.671 | 0.000 ± 0.810 | –0.200 | 0.421 |
|  |  |  |  |  |  |
| G7 | T1 | 0.001 ± 0.586 | 0.000 ± 0.816 | 0.007 | 0.497 |
|  | T2 | 0.010 ± 0.546 | 0.003 ± 0.863 | 0.108 | 0.457 |
|  | T3 | 0.001 ± 0.584 | 0.004 ± 0.876 | –0.043 | 0.483 |
|  | T4 | –0.015 ± 0.666 | 0.000 ± 0.842 | –0.218 | 0.414 |
|  | T5 | –0.002 ± 0.59 | 0.006 ± 0.845 | –0.136 | 0.446 |
|  |  |  |  |  |  |
| G9 | T1 | 0.004 ± 0.543 | –0.005 ± 0.802 | 0.177 | 0.430 |
|  | T2 | –0.001 ± 0.638 | 0.005 ± 0.856 | –0.095 | 0.462 |
|  | T3 | –0.006 ± 0.642 | –0.003 ± 0.808 | –0.039 | 0.485 |
|  | T4 | 0.002 ± 0.598 | –0.011 ± 0.868 | 0.175 | 0.431 |
|  | T5 | –0.004 ± 0.554 | –0.002 ± 0.779 | –0.026 | 0.490 |

**Table S5**

*The confirmatory factor analysis results of executive functions of three grade cohorts and across timewaves*

| Cohort | Wave | model | *χ^2^* | Scaling | | | CFI | TLI | SRMR | RMSEA (90% CI) | ΔCFI | ΔRMSEA |
| --- | --- | --- | --- | --- | --- | --- | --- | --- | --- | --- | --- | --- |
| G5 |  |  |  |  | | |  |  |  |  |  |  |
|  | T1 | M1 | 56.29 | 0.89 | | | .812 | .737 | .056 | .080 [.056, .105] |  |  |
|  |  | **M2a** | **26.99** | **0.92** | | | **.959** | **.939** | **.043** | **.038 [.000, .069]** |  |  |
|  |  | M2b | 57.18 | 0.87 | | | .802 | .709 | .056 | .084 [.059, .109] |  |  |
|  |  | M2c | 55.53 | 0.88 | | | .811 | .721 | .055 | .082 [.058, .108] |  |  |
|  |  | M3 | 26.64 | 0.90 | | | .950 | .918 | .042 | .045 [.000, .076] |  |  |
|  |  | M2a vs M1 |  |  | |  | |  |  |  | .147 | .042 |
|  |  | M2b vs M1 |  |  | |  | |  |  |  | .010 | .004 |
|  |  | M2c vs M1 |  |  | |  | |  |  |  | .001 | .002 |
|  |  | M3 vs M2a |  |  | |  | |  |  |  | .009 | .007 |
|  |  |  |  |  | | |  |  |  |  |  |  |
|  | T2 | M1 | 65.21 | 1.01 | | | .821 | .749 | .066 | .089 [.066, .114] |  |  |
|  |  | M2a | 39.70 | 0.99 | | | .918 | .879 | .065 | .062 [.034, .089] |  |  |
|  |  | M2b | 60.48 | 1.00 | | | .836 | .758 | .066 | .088 [.063, .113] |  |  |
|  |  | M2c | 52.16 | 1.01 | | | .869 | .806 | .060 | .078 [.053, .104] |  |  |
|  |  | **M3** | **29.20** | **0.95** | | | **.952** | **.920** | **.058** | **.050 [.014, .080]** |  |  |
|  |  | M2a vs M1 |  |  | |  | |  |  |  | .097 | .027 |
|  |  | M2b vs M1 |  |  | |  | |  |  |  | .015 | .001 |
|  |  | M2c vs M1 |  |  | |  | |  |  |  | .048 | .011 |
|  |  | M3 vs M2a |  |  | |  | |  |  |  | .034 | .012 |
|  |  |  |  |  | | |  |  |  |  |  |  |
|  | T3 | M1 | 95.88 | 1.00 | | | .798 | .717 | .079 | .116 [.093, .140] |  |  |
|  |  | M2a | 47.98 | 1.03 | | | .923 | .886 | .059 | .073 [.048, .100] |  |  |
|  |  | M2b | 99.47 | 0.95 | | | .786 | .685 | .078 | .122 [.099, .147] |  |  |
|  |  | M2c | 82.03 | 0.95 | | | .832 | .753 | .071 | .108 [.085, .133] |  |  |
|  |  | **M3** | **31.41** | **1.02** | | | **.962** | **.937** | **.048** | **.055 [.022, .084]** |  |  |
|  |  | M2a vs M1 |  |  | |  | |  |  |  | .125 | .043 |
|  |  | M2b vs M1 |  |  | |  | |  |  |  | .012 | .006 |
|  |  | M2c vs M1 |  |  | |  | |  |  |  | .034 | .008 |
|  |  | M3 vs M2a |  |  | |  | |  |  |  | .039 | .018 |
|  |  |  |  |  | | |  |  |  |  |  |  |
|  | T4 | M1 | 66.72 | 1.06 | | | .857 | .799 | .062 | .091 [.067, .115] |  |  |
|  |  | M2a | 42.74 | 1.05 | | | .927 | .893 | .056 | .066 [.040, .093] |  |  |
|  |  | M2b | 60.28 | 1.02 | | | .873 | .813 | .060 | .088 [.063, .113] |  |  |
|  |  | M2c | 50.89 | 1.04 | | | .902 | .856 | .055 | .077 [.052, .103] |  |  |
|  |  | **M3** | **28.18** | **1.01** | | | **.966** | **.943** | **.049** | **.048 [.009, .079]** |  |  |
|  |  | M2a vs M1 | | | | | |  |  |  | .070 | .025 |
|  |  | M2b vs M1 | | | | | |  |  |  | .016 | .003 |
|  |  | M2c vs M1 | | | | | |  |  |  | .045 | .014 |
|  |  | M3 vs M2a | | | | | |  |  |  | .039 | .018 |
|  |  |  | | | | | |  |  |  |  |  |
|  | T5 | M1 | 60.23 | 1.02 | | | .873 | .822 | .060 | .087 [.062, .112] |  |  |
|  |  | M2a | 45.16 | 0.98 | | | .917 | .878 | .052 | .072 [.045, .099] |  |  |
|  |  | M2b* | 63.64 | 0.96 | | | .859 | .792 | .060 | .094 [.069, .120] |  |  |
|  |  | M2c | 41.06 | 0.98 | | | .930 | .897 | .050 | .066 [.038, .094] |  |  |
|  |  | **M3** | **20.48** | **0.99** | | | **.989** | **.982** | **.036** | **.028 [.000, .065]** |  |  |
|  |  | M2a vs M1 |  |  | |  | |  |  |  | .044 | .015 |
|  |  | M2b vs M1 |  |  | |  | |  |  |  | .014 | .007 |
|  |  | M2c vs M1 |  |  | |  | |  |  |  | .057 | .021 |
|  |  | M3 vs M2c |  |  | |  | |  |  |  | .059 | .038 |
|  |  |  |  | | | | |  |  |  |  |  |
| **G7** |  |  |  | |  | |  |  |  |  |  |  |
|  | T1 | M1 | 60.64 | 0.81 | | | .624 | .473 | .088 | .129 [.092, .166] |  |  |
|  |  | M2a | 39.70 | 0.89 | | | .808 | .717 | .077 | .094 [.052, .135] |  |  |
|  |  | M2b | 50.82 | 0.84 | | | .705 | .566 | .086 | .117 [.078, .156] |  |  |
|  |  | M2c | 29.59 | 0.91 | | | .902 | .856 | .058 | .067 [.000, .112] |  |  |
|  |  | **M3** | **21.99** | **0.91** | | | **.954** | **.924** | **.046** | **.049 [.000, .101]** |  |  |
|  |  | M2a vs M1 |  | | | | |  |  |  | .184 | .035 |
|  |  | M2b vs M1 |  | | | | |  |  |  | .081 | .012 |
|  |  | M2c vs M1 |  | | | | |  |  |  | .278 | .062 |
|  |  | M3 vs M2c |  | | | | |  |  |  | .052 | .018 |
|  |  |  |  | | | | |  |  |  |  |  |
|  | T2 | M1 | 92.81 | 0.90 | | | .530 | .342 | .082 | .119 [.096, .145] |  |  |
|  |  | M2a | 37.10 | 0.92 | | | .883 | .828 | .050 | .061 [.031, .090] |  |  |
|  |  | M2b | 513.94 | 0.17 | | | .000 | -3.71 | .081 | .320 [.296, .344] |  |  |
|  |  | M2c | 201.35 | 0.36 | | | .000 | -0.73 | .074 | .194 [.170, .219] |  |  |
|  |  | **M3** | **24.16** | **0.97** | | | **.954** | **.924** | **.039** | **.041 [.000, .075]** |  |  |
|  |  | M2a vs M1 |  |  | | |  |  |  |  | .353 | .058 |
|  |  | M3 vs M2a |  | | | | |  |  |  | .071 | .020 |
|  |  |  |  | | | | |  |  |  |  |  |
|  | T3 | M1 | 137.75 | 0.90 | | | .490 | .285 | .096 | .152 [.129, .176] |  |  |
|  |  | M2a | 44.85 | 0.94 | | | .888 | .835 | .060 | .073 [.045, .101] |  |  |
|  |  | M2b | 145.56 | 0.85 | | | .451 | .191 | .096 | .162 [.138, .187] |  |  |
|  |  | M2c | 127.48 | 0.84 | | | .530 | .307 | .087 | .150 [.126, .175] |  |  |
|  |  | **M3** | **25.60** | **0.97** | | | **.963** | **.939** | **.042** | **.045 [.000, .078]** |  |  |
|  |  | M2a vs M1 |  |  | | |  |  |  |  | .398 | .079 |
|  |  | M2b vs M1 |  |  | | |  |  |  |  | .039 | .010 |
|  |  | M2c vs M1 |  |  | | |  |  |  |  | .040 | .002 |
|  |  | M3 vs M2a |  |  | | |  |  |  |  | .075 | .028 |
|  |  |  |  |  | | |  |  |  |  |  |  |
|  | T4 | M1 | 86.53 | 0.94 | | | .722 | .610 | .088 | .128 [.101, .157] |  |  |
|  |  | M2a | 41.20 | 0.95 | | | .907 | .863 | .068 | .076 [.044, .108] |  |  |
|  |  | M2b | 94.05 | 0.84 | | | .686 | .537 | .087 | .140 [.112, .169] |  |  |
|  |  | M2c | 85.32 | 0.88 | | | .723 | .591 | .085 | .131 [.104, .160] |  |  |
|  |  | **M3** | **30.90** | **0.91** | | | **.942** | **.904** | **.061** | **.064 [.025, .099]** |  |  |
|  |  | M2a vs M1 |  |  | | |  |  |  |  | .185 | .052 |
|  |  | M2b vs M1 |  |  | | |  |  |  |  | .036 | .012 |
|  |  | M2c vs M1 |  |  | | |  |  |  |  | .001 | .003 |
|  |  | M3 vs M2a |  |  | | |  |  |  |  | .035 | .012 |
|  |  |  |  |  | | |  |  |  |  |  |  |
|  | T5 | M1 | 81.05 | 1.03 | | | .665 | .531 | .082 | .104 [.081, .129] |  |  |
|  |  | M2a | 20.70 | 1.09 | | | .991 | .986 | .044 | .018 [.000, .057] |  |  |
|  |  | M2b | 84.18 | 0.99 | | | .642 | .473 | .081 | .111 [.087, .135] |  |  |
|  |  | M2c | 73.54 | 1.04 | | | .701 | .559 | .075 | .101 [.077, .126] |  |  |
|  |  | **M3** | **11.91** | **1.07** | | | **1.000** | **1.046** | **.032** | **.000 [.000, .035]** |  |  |
|  |  | M2a vs M1 |  |  | | |  |  |  |  | .326 | .086 |
|  |  | M2b vs M1 |  |  | | |  |  |  |  | .023 | .007 |
|  |  | M2c vs M1 |  |  | | |  |  |  |  | .036 | .003 |
|  |  | M3 vs M2a |  |  | | |  |  |  |  | .009 | .018 |
| **G9** |  |  |  |  | | |  |  |  |  |  |  |
|  | T1 | M1 | 54.94 | 0.88 | | | .762 | .666 | .056 | .081 [.056, .107] |  |  |
|  |  | M2a | 38.14 | 0.87 | | | .869 | .808 | .045 | .062 [.032, .090] |  |  |
|  |  | M2b | 50.56 | 0.86 | | | .785 | .683 | .053 | .079 [.053, .106] |  |  |
|  |  | M2c | 40.65 | 0.85 | | | .852 | .782 | .046 | .066 [.037, .094] |  |  |
|  |  | **M3** | **24.39** | **0.86** | | | **.950** | **.917** | **.035** | **.041 [.000, .074]** |  |  |
|  |  | M2a vs M1 |  |  | | |  |  |  |  | .107 | .019 |
|  |  | M2b vs M1 |  |  | | |  |  |  |  | .023 | .002 |
|  |  | M2c vs M1 |  |  | | |  |  |  |  | .090 | .015 |
|  |  | M3 vs M2a |  |  | | |  |  |  |  | .081 | .021 |
|  |  |  |  |  | | |  |  |  |  |  |  |
|  | T2 | M1 | 59.55 | 0.97 | | | .823 | .752 | .069 | .088 [.063, .115] |  |  |
|  |  | M2a | 23.24 | 0.96 | | | .981 | .972 | .042 | .030 [.000, .065] |  |  |
|  |  | M2b | 63.06 | 0.90 | | | .803 | .710 | .096 | .096 [.070, .122] |  |  |
|  |  | M2c | 57.61 | 0.89 | | | .827 | .746 | .065 | .089 [.064, .116] |  |  |
|  |  | **M3** | **18.16** | **0.91** | | | **.995** | **.991** | **.035** | **.016 [.000, .060]** |  |  |
|  |  | M2a vs M1 |  |  | | |  |  |  |  | .158 | .058 |
|  |  | M2b vs M1 |  |  | | |  |  |  |  | .020 | .008 |
|  |  | M2c vs M1 |  |  | | |  |  |  |  | .004 | .001 |
|  |  | M3 vs M2a |  |  | | |  |  |  |  | .014 | .014 |
|  |  |  |  |  | | |  |  |  |  |  |  |
|  | T3 | M1 | 45.18 | 1.21 | | | .847 | .785 | .062 | .082 [.050, .114] |  |  |
|  |  | **M2a** | **34.50** | **1.22** | | | **.906** | **.861** | **.053** | **.066 [.028, .101]** |  |  |
|  |  | M2b | 45.09 | 1.21 | | | .841 | .766 | .062 | .086 [.054, .118] |  |  |
|  |  | M2c | 48.90 | 1.03 | | | .818 | .731 | .059 | .092 [.060, .124] |  |  |
|  |  | M3 | 33.72 | 1.07 | | | .898 | .832 | .048 | .073 [.035, .108] |  |  |
|  |  | M2a vs M1 |  |  | | |  |  |  |  | .059 | .016 |
|  |  | M2b vs M1 |  |  | | |  |  |  |  | .006 | .004 |
|  |  | M2c vs M1 |  |  | | |  |  |  |  | .029 | .010 |
|  |  | M3 vs M2a |  |  | | |  |  |  |  | .008 | .007 |
|  |  |  |  |  | | |  |  |  |  |  |  |
|  | T4 | M1 | 31.76 | 1.00 | | | .889 | .844 | .059 | .058 [.008, .095] |  |  |
|  |  | **M2a** | **10.92** | **0.99** | | | **1.000** | **1.113** | **.035** | **.000 [.000, .021]** |  |  |
|  |  | M2b* | 34.89 | 0.91 | | | .850 | .778 | .059 | .069 [.031, .105] |  |  |
|  |  | M2c* | 36.29 | 0.87 | | | .836 | .759 | .059 | .072 [.035, .108] |  |  |
|  |  | M3* | 12.31 | 0.82 | | | 1.000 | 1.073 | .034 | .000 [.000, .047] |  |  |
|  |  | M2a vs M1 |  |  | | |  |  |  |  | .111 | .058 |
|  |  |  |  |  | | |  |  |  |  |  |  |
|  | T5 | M1 | 19.59 | 0.96 | | | 1.000 | 1.011 | .044 | .000 [.000, .066] |  |  |
|  |  | **M2a** | **18.32** | **0.94** | | | **1.000** | **1.019** | **.042** | **.000 [.000, .066]** |  |  |
|  |  | M2b | 19.25 | 0.96 | | | .995 | .993 | .043 | .009 [.000, .070] |  |  |
|  |  | M2c | 19.84 | 0.95 | | | .984 | .976 | .044 | .017 [.000, .072] |  |  |
|  |  | M3 | 19.23 | 0.89 | | | .957 | .929 | .043 | .029 [.000, .079] |  |  |
|  |  | M2a vs M1 |  |  | | |  |  |  |  | .000 | .000 |
|  |  | M2b vs M1 |  |  | | |  |  |  |  | .005 | .009 |
|  |  | M2c vs M1 |  |  | | |  |  |  |  | .016 | .017 |
|  |  | M3 vs M2a |  |  | | |  |  |  |  | .043 | .029 |

M1 = single-factor model; M2a = inhibition-switching and updating model; M2b = inhibition-updating and switching model; M2c = switching -updating and inhibition model; M3 = three-factor model. The *df*s for M1, M2a, M2b, M2c, and M3 were 20, 19, 19, 19, and 17, respectively.

* Not positive definite residual covariance matrix.

**Table S6**

*Longitudinal measurement equivalence test*

| Model | *χ*^2^ | *df* | scaling | CFI | TLI | RMSEA | ΔCFI |
| --- | --- | --- | --- | --- | --- | --- | --- |
| **G5 T2 to T5** |  |  |  |  |  |  |  |
| **Configural** | **442.73** | **350** | **0.99** | **.964** | **.950** | **.030 [.021, .039]** |  |
| metric | 481.46 | 365 | 1.00 | .955 | .939 | .033 [.025, .041] | 0.009 |
| scalar | 730.91 | 389 | 1.01 | .869 | .833 | .055 [.049, .061] | 0.086 |
|  |  |  |  |  |  |  |  |
| **G7 T1 to T5** |  |  |  |  |  |  |  |
| configural | 765.43 | 555 | 0.97 | .901 | .861 | .035 [.029, .041] |  |
| **metric** | **802.33** | **575** | **0.99** | **.893** | **.855** | **.036 [.030, .042]** | **0.008** |
| scalar | 1032.18 | 607 | 0.99 | .800 | .743 | .048 [.043, .053] | 0.093 |
|  |  |  |  |  |  |  |  |
| **G9 T1 to T2** |  |  |  |  |  |  |  |
| configural | 105.77 | 81 | 0.92 | .959 | .940 | .034 [.010, .051] |  |
| **metric** | **111.72** | **86** | **0.94** | **.958** | **.941** | **.034 [.010, .050]** | **0.001** |
| scalar | 149.88 | 94 | 0.94 | .908 | .883 | .047 [.033, .061] | 0.050 |
|  |  |  |  |  |  |  |  |
| **G9 T3 to T5** |  |  |  |  |  |  |  |
| configural | 353.52 | 213 | 0.88 | .830 | .780 | .058 [.047, .069] |  |
| metric | 365.66 | 225 | 0.92 | .830 | .791 | .056 [.046, .067] | 0.000 |
| scalar | 414.64 | 241 | 0.92 | .790 | .760 | .061 [.051, .070] | 0.040 |

**Table S7**

*Measurement equivalence test across cohorts*

| Model | *χ*^2^ | *df* | scaling | CFI | TLI | RMSEA | ΔCFI |
| --- | --- | --- | --- | --- | --- | --- | --- |
| **T1 G5 and T3 G9** |  |  |  |  |  |  |  |
| **Configural** | **62.56** | **38** | **1.07** | **.931** | **.898** | **.052 [.027, .075]** |  |
| metric | 76.47 | 44 | 1.16 | .908 | .883 | .056 [.034, .076] | 0.023 |
| scalar | 104.82 | 50 | 1.14 | .845 | .826 | .068 [.050, .086] | 0.063 |
|  |  |  |  |  |  |  |  |
| **T1 G5 and T4 G9** |  |  |  |  |  |  |  |
| **configural** | **37.33** | **38** | **0.95** | **1.000** | **1.003** | **.000 [.000, .045]** |  |
| metric | 77.19 | 44 | 0.96 | .885 | .854 | .057 [.035, .078] | 0.115 |
| scalar | 99.19 | 50 | 1.05 | .830 | .809 | .065 [.046, .084] | 0.055 |
|  |  |  |  |  |  |  |  |
| **T1 G5 and T5 G9** |  |  |  |  |  |  |  |
| **configural** | **45.21** | **38** | **0.93** | **.969** | **.954** | **.029 [.000, .058]** |  |
| metric | 73.22 | 44 | 0.94 | .873 | .838 | .055 [.031, .076] | 0.096 |
| scalar | 103.45 | 50 | 0.98 | .767 | .739 | .069 [.050, .088] | 0.106 |

**Table S8**

*The results of exploratory factor analysis*

|  |  | G5 | | |  | G7 | | |  | G9 | | |
| --- | --- | --- | --- | --- | --- | --- | --- | --- | --- | --- | --- | --- |
|  |  | IS | U |  |  | I | S | U |  | I | S | U |
| T1 | Flanker | 0.48 |  |  |  | 0.84 |  |  |  | 0.80 |  |  |
|  | Stroop | 0.72 |  |  |  | 0.89 |  |  |  | 0.76 |  |  |
|  | PS | 0.67 |  |  |  |  | 0.26 |  |  |  | 0.36 |  |
|  | DCCS | 0.55 |  |  |  |  | 0.81 |  |  |  | 0.66 |  |
|  | WCST | 0.41 |  |  |  |  | 0.59 |  |  |  | 0.80 |  |
|  | VM |  | 0.14 |  |  |  |  | 0.27 |  |  |  | 0.67 |
|  | Rank |  | 0.77 |  |  |  |  | 0.81 |  |  |  | 0.75 |
|  | BDST |  | 0.83 |  |  |  |  | 0.70 |  |  |  | 0.65 |
|  |  |  |  |  |  |  |  |  |  |  |  |  |
|  |  | I | S | U |  | I | S | U |  | I | S | U |
| T2 | Flanker | 0.79 |  |  |  | 0.64 |  |  |  | 0.69 |  |  |
|  | Stroop | 0.79 |  |  |  | 0.74 |  |  |  | 0.79 |  |  |
|  | PS |  | 0.67 |  |  |  | 0.47 |  |  |  | 0.71 |  |
|  | DCCS |  | 0.60 |  |  |  | 0.66 |  |  |  | 0.51 |  |
|  | WCST |  | 0.39 |  |  |  | 0.65 |  |  |  | 0.68 |  |
|  | VM |  |  | 0.93 |  |  |  | 0.72 |  |  |  | 0.94 |
|  | Rank |  |  | 0.81 |  |  |  | 0.82 |  |  |  | 0.79 |
|  | BDST |  |  | 0.83 |  |  |  | 0.81 |  |  |  | 0.88 |
|  |  |  |  |  |  |  |  |  |  |  |  |  |
|  |  | I | S | U |  | I | S | U |  | IS | U |  |
| T3 | Flanker | 0.81 |  |  |  | 0.80 |  |  |  | 0.68 |  |  |
|  | Stroop | 0.66 |  |  |  | 0.78 |  |  |  | 0.75 |  |  |
|  | PS |  | 0.59 |  |  |  | 0.23 |  |  | 0.62 |  |  |
|  | DCCS |  | 0.25 |  |  |  | 0.79 |  |  | 0.44 |  |  |
|  | WCST |  | 0.81 |  |  |  | 0.76 |  |  | 0.63 |  |  |
|  | VM |  |  | 0.52 |  |  |  | 0.38 |  |  | 0.63 |  |
|  | Rank |  |  | 0.81 |  |  |  | 0.85 |  |  | 0.74 |  |
|  | BDST |  |  | 0.79 |  |  |  | 0.85 |  |  | 0.68 |  |
|  |  |  |  |  |  |  |  |  |  |  |  |  |
|  |  | I | S | U |  | I | S | U |  | IS | U |  |
| T4 | Flanker | 0.73 |  |  |  | 0.80 |  |  |  | 0.49 |  |  |
|  | Stroop | 0.79 |  |  |  | 0.73 |  |  |  | 0.70 |  |  |
|  | PS |  | 0.71 |  |  |  | 0.62 |  |  | 0.67 |  |  |
|  | DCCS |  | 0.53 |  |  |  | 0.45 |  |  | 0.57 |  |  |
|  | WCST |  | 0.68 |  |  |  | 0.88 |  |  | 0.72 |  |  |
|  | VM |  |  | 0.98 |  |  |  | 0.61 |  |  | 0.41 |  |
|  | Rank |  |  | 0.82 |  |  |  | 0.80 |  |  | 0.81 |  |
|  | BDST |  |  | 0.80 |  |  |  | 0.75 |  |  | 0.78 |  |
|  |  |  |  |  |  |  |  |  |  |  |  |  |
|  |  | I | S | U |  | I | S | U |  | IS | U |  |
| T5 | Flanker | 0.77 |  |  |  | 0.83 |  |  |  | 0.18 |  |  |
|  | Stroop | 0.81 |  |  |  | 0.71 |  |  |  | 0.66 |  |  |
|  | PS |  | 0.56 |  |  |  | 0.44 |  |  | 0.62 |  |  |
|  | DCCS |  | 0.65 |  |  |  | 0.76 |  |  | 0.66 |  |  |
|  | WCST |  | 0.55 |  |  |  | 0.69 |  |  | 0.62 |  |  |
|  | VM |  |  | 0.94 |  |  |  | 0.55 |  |  | 0.18 |  |
|  | Rank |  |  | 0.78 |  |  |  | 0.77 |  |  | 0.79 |  |
|  | BDST |  |  | 0.79 |  |  |  | 0.81 |  |  | 0.70 |  |

*Note.* Flanker = Flanker task; Stroop = Stroop task; PS = Picture Symbol task; DCCS = Dimension Change Card Sort task; WCST = Wisconsin Card Sorting task; VM = Visuospatial Memory task; Rank = 2-List List Sorting; BDST = Backwards Digit Span task.

Cross-loading items were assessed by examining the difference between their primary and secondary factor loadings. A minimum difference of ≥ 0.20 was required to ensure adequate discriminant validity, and only the primary factor loading was retained (Howard, 2016).

Reference

Howard, M. C. (2016). A review of exploratory factor analysis decisions and overview of current practices: What we are doing and how can we improve? *International Journal of Human–Computer Interaction, 32*(1), 51–62. https://doi.org/10.1080/10447318.2015.1087664

**Table S9**

*The confirmatory factor analysis results of executive functions of three grade cohorts and across timewaves (multiple imputation)*

| Cohort | Wave | model | *χ^2^* | Scaling | | | CFI | TLI | SRMR | RMSEA (90% CI) | ΔCFI | ΔRMSEA |
| --- | --- | --- | --- | --- | --- | --- | --- | --- | --- | --- | --- | --- |
| G5 |  |  |  |  | | |  |  |  |  |  |  |
|  | T1 | M1 | 103.53 | 0.96 | | | .823 | .752 | .057 | .083 [.066, .098] |  |  |
|  |  | **M2a** | **59.69** | **0.95** | | | **.914** | **.873** | **.044** | **.059 [.041, .075]** |  |  |
|  |  | M2b | 100.66 | 0.94 | | | .827 | .745 | .056 | .084 [.066, .098] |  |  |
|  |  | M2c | 85.61 | 0.92 | | | .859 | .792 | .052 | .076 [.057, .090] |  |  |
|  |  | M3 | 172.43 | 0.89 | | | .909 | .881 | .042 | .072 [.065, .085] |  |  |
|  |  | M2a vs M1 |  |  | |  | |  |  |  | .091 | .024 |
|  |  | M2b vs M1 |  |  | |  | |  |  |  | .004 | .001 |
|  |  | M2c vs M1 |  |  | |  | |  |  |  | .036 | .007 |
|  |  | M3 vs M2a |  |  | |  | |  |  |  | .005 | .013 |
|  |  |  |  |  | | |  |  |  |  |  |  |
|  | T2 | M1 | 142.84 | 1.05 | | | .792 | .709 | .073 | .100 [.088, .118] |  |  |
|  |  | M2a | 56.50 | 1.02 | | | .937 | .907 | .050 | .057 [.041, .075] |  |  |
|  |  | M2b | 150.58 | 0.95 | | | .778 | .672 | .073 | .106 [.088, .120] |  |  |
|  |  | M2c | 119.37 | 1.00 | | | .830 | .759 | .066 | .093 [.077, .109] |  |  |
|  |  | **M3** | **30.23** | **0.98** | | | **.978** | **.963** | **.041** | **.036 [.012, .056]** |  |  |
|  |  | M2a vs M1 |  |  | |  | |  |  |  | .145 | .043 |
|  |  | M2b vs M1 |  |  | |  | |  |  |  | .014 | .006 |
|  |  | M2c vs M1 |  |  | |  | |  |  |  | .038 | .007 |
|  |  | M3 vs M2a |  |  | |  | |  |  |  | .041 | .019 |
|  |  |  |  |  | | |  |  |  |  |  |  |
|  | T3 | M1 | 199.15 | 1.09 | | | .751 | .652 | .081 | .117 [.108, .137] |  |  |
|  |  | M2a | 88.78 | 1.10 | | | .903 | .857 | .055 | .075 [.063, .095] |  |  |
|  |  | M2b | 206.26 | 1.04 | | | .740 | .617 | .081 | .123 [.111, .141] |  |  |
|  |  | M2c | 161.05 | 1.06 | | | .803 | .709 | .073 | .107 [.095, .126] |  |  |
|  |  | **M3** | **45.14** | **1.10** | | | **.961** | **.936** | **.042** | **.050 [.035, .071]** |  |  |
|  |  | M2a vs M1 |  |  | |  | |  |  |  | .152 | .042 |
|  |  | M2b vs M1 |  |  | |  | |  |  |  | .011 | .006 |
|  |  | M2c vs M1 |  |  | |  | |  |  |  | .052 | .010 |
|  |  | M3 vs M2a |  |  | |  | |  |  |  | .058 | .025 |
|  |  |  |  |  | | |  |  |  |  |  |  |
|  | T4 | M1 | 142.33 | 1.07 | | | .816 | .743 | .072 | .100 [.088, .119] |  |  |
|  |  | M2a | 72.10 | 1.06 | | | .920 | .882 | .056 | .068 [.054, .087] |  |  |
|  |  | M2b | 146.03 | 0.97 | | | .809 | .719 | .073 | .105 [.088, .119] |  |  |
|  |  | M2c | 116.36 | 1.02 | | | .854 | .784 | .066 | .092 [.077, .109] |  |  |
|  |  | **M3** | **39.11** | **1.01** | | | **.967** | **.945** | **.047** | **.046 [.027, .066]** |  |  |
|  |  | M2a vs M1 | | | | | |  |  |  | .104 | .032 |
|  |  | M2b vs M1 | | | | | |  |  |  | .007 | .005 |
|  |  | M2c vs M1 | | | | | |  |  |  | .038 | .008 |
|  |  | M3 vs M2a | | | | | |  |  |  | .047 | .022 |
|  |  |  | | | | | |  |  |  |  |  |
|  | T5 | M1 | 116.71 | 1.07 | | | .832 | .765 | .062 | .086 [.074, .104] |  |  |
|  |  | M2a | 56.75 | 1.07 | | | .935 | .904 | .042 | .055 [.041, .074] |  |  |
|  |  | M2b | 126.24 | 0.96 | | | .814 | .726 | .063 | .093 [.076, .107] |  |  |
|  |  | M2c | 83.79 | 1.04 | | | .888 | .834 | .054 | .072 [.058, .089] |  |  |
|  |  | **M3** | **19.90** | **1.04** | | | **.995** | **.992** | **.028** | **.016 [.000, .041]** |  |  |
|  |  | M2a vs M1 |  |  | |  | |  |  |  | .103 | .031 |
|  |  | M2b vs M1 |  |  | |  | |  |  |  | .018 | .007 |
|  |  | M2c vs M1 |  |  | |  | |  |  |  | .056 | .014 |
|  |  | M3 vs M2c |  |  | |  | |  |  |  | .060 | .039 |
|  |  |  |  | | | | |  |  |  |  |  |
| **G7** |  |  |  | |  | |  |  |  |  |  |  |
|  | T1 | M1 | 44.59 | 0.90 | | | .749 | .648 | .091 | .097 [.059, .136] |  |  |
|  |  | M2a | 30.57 | 0.93 | | | .882 | .826 | .077 | .070 [.011, .113] |  |  |
|  |  | M2b | 37.91 | 0.87 | | | .807 | .715 | .084 | .086 [.045, .126] |  |  |
|  |  | M2c | 30.93 | 0.82 | | | .878 | .820 | .068 | .066 [.014, .107] |  |  |
|  |  | **M3** | **18.07** | **0.90** | | | **.989** | **.982** | **.050** | **.022 [.000, .084]** |  |  |
|  |  | M2a vs M1 |  | | | | |  |  |  | .133 | .027 |
|  |  | M2b vs M1 |  | | | | |  |  |  | .058 | .011 |
|  |  | M2c vs M1 |  | | | | |  |  |  | .129 | .031 |
|  |  | M3 vs M2c |  | | | | |  |  |  | .107 | .048 |
|  |  |  |  | | | | |  |  |  |  |  |
|  | T2 | M1 | 78.23 | 0.96 | | | .544 | .361 | .088 | .115 [.088, .142] |  |  |
|  |  | M2a | 32.56 | 0.93 | | | .894 | .843 | .053 | .056 [.019, .088] |  |  |
|  |  | M2b | 68.47 | 0.99 | | | .612 | .429 | .094 | .110 [.082, .138] |  |  |
|  |  | M2c | 204.75 | 0.30 | | | .430 | .450 | .078 | .117 [.103, .132] |  |  |
|  |  | **M3** | **19.78** | **0.97** | | | **.978** | **.964** | **.039** | **.027 [.000, .070]** |  |  |
|  |  | M2a vs M1 |  |  | | |  |  |  |  | .350 | .059 |
|  |  | M2b vs M1 |  |  | | |  |  |  |  | .068 | .005 |
|  |  | M2c vs M1 |  |  | | |  |  |  |  | .114 | .002 |
|  |  | M3 vs M2c |  | | | | |  |  |  | .084 | .029 |
|  |  |  |  | | | | |  |  |  |  |  |
|  | T3 | M1 | 116.89 | 0.91 | | | .521 | .330 | .099 | .140 [.116, .165] |  |  |
|  |  | M2a | 41.71 | 0.94 | | | .888 | .835 | .066 | .071 [.041, .100] |  |  |
|  |  | M2b | 121.06 | 0.88 | | | .496 | .257 | .099 | .145 [.121, .170] |  |  |
|  |  | M2c | 104.81 | 0.87 | | | .576 | .375 | .091 | .132 [.108, .158] |  |  |
|  |  | **M3** | **25.03** | **0.97** | | | **.960** | **.935** | **.050** | **.045 [.000, .080]** |  |  |
|  |  | M2a vs M1 |  |  | | |  |  |  |  | .367 | .069 |
|  |  | M2b vs M1 |  |  | | |  |  |  |  | .025 | .005 |
|  |  | M2c vs M1 |  |  | | |  |  |  |  | .055 | .008 |
|  |  | M3 vs M2a |  |  | | |  |  |  |  | .072 | .026 |
|  |  |  |  |  | | |  |  |  |  |  |  |
|  | T4 | M1 | 80.68 | 1.12 | | | .699 | .579 | .094 | .120 [.094, .148] |  |  |
|  |  | M2a | 38.45 | 0.90 | | | .904 | .858 | .067 | .070 [.037, .101] |  |  |
|  |  | M2b | 90.63 | 0.79 | | | .645 | .477 | .094 | .125 [.100, .152] |  |  |
|  |  | M2c | 77.20 | 0.86 | | | .712 | .575 | .091 | .118 [.091, .145] |  |  |
|  |  | **M3** | **25.27** | **0.89** | | | **.959** | **.932** | **.058** | **.048 [.000, .085]** |  |  |
|  |  | M2a vs M1 |  |  | | |  |  |  |  | .205 | .050 |
|  |  | M2b vs M1 |  |  | | |  |  |  |  | .054 | .005 |
|  |  | M2c vs M1 |  |  | | |  |  |  |  | .013 | .002 |
|  |  | M3 vs M2a |  |  | | |  |  |  |  | .055 | .022 |
|  |  |  |  |  | | |  |  |  |  |  |  |
|  | T5 | M1 | 81.26 | 0.99 | | | .651 | .512 | .082 | .108 [.084, .133] |  |  |
|  |  | M2a | 24.96 | 1.03 | | | .966 | .950 | .044 | .035 [.000, .069] |  |  |
|  |  | M2b | 86.30 | 0.93 | | | .617 | .435 | .082 | .113 [.089, .137] |  |  |
|  |  | M2c | 71.92 | 0.98 | | | .699 | .556 | .076 | .103 [.078, .128] |  |  |
|  |  | **M3** | **13.41** | **1.03** | | | **1.000** | **1.000** | **.033** | **.000 [.000, .044]** |  |  |
|  |  | M2a vs M1 |  |  | | |  |  |  |  | .315 | .073 |
|  |  | M2b vs M1 |  |  | | |  |  |  |  | .034 | .005 |
|  |  | M2c vs M1 |  |  | | |  |  |  |  | .048 | .005 |
|  |  | M3 vs M2a |  |  | | |  |  |  |  | .034 | .035 |
| **G9** |  |  |  |  | | |  |  |  |  |  |  |
|  | T1 | M1 | 56.22 | 0.88 | | | .750 | .650 | .063 | .081 [.056, .106] |  |  |
|  |  | M2a | 39.78 | 0.87 | | | .857 | .789 | .052 | .063 [.035, .090] |  |  |
|  |  | M2b | 51.44 | 0.85 | | | .776 | .670 | .060 | .077 [.052, .103] |  |  |
|  |  | M2c | 42.93 | 0.84 | | | .835 | .757 | .053 | .066 [.040, .092] |  |  |
|  |  | **M3** | **26.35** | **0.86** | | | **.935** | **.894** | **.041** | **.044 [.000, .075]** |  |  |
|  |  | M2a vs M1 |  |  | | |  |  |  |  | .107 | .018 |
|  |  | M2b vs M1 |  |  | | |  |  |  |  | .026 | .004 |
|  |  | M2c vs M1 |  |  | | |  |  |  |  | .085 | .015 |
|  |  | M3 vs M2a |  |  | | |  |  |  |  | .078 | .019 |
|  |  |  |  |  | | |  |  |  |  |  |  |
|  | T2 | M1 | 52.28 | 1.00 | | | .819 | .747 | .076 | .097 [.065, .129] |  |  |
|  |  | M2a | 20.95 | 0.98 | | | .979 | .974 | .049 | .034 [.000, .072] |  |  |
|  |  | M2b | 55.84 | 0.94 | | | .794 | .696 | .076 | .102 [.072, .134] |  |  |
|  |  | M2c | 52.62 | 0.90 | | | .812 | .723 | .072 | .096 [.066, .127] |  |  |
|  |  | **M3** | **17.73** | **0.91** | | | **.996** | **.993** | **.041** | **.015 [.000, .068]** |  |  |
|  |  | M2a vs M1 |  |  | | |  |  |  |  | .160 | .063 |
|  |  | M2b vs M1 |  |  | | |  |  |  |  | .025 | .005 |
|  |  | M2c vs M1 |  |  | | |  |  |  |  | .007 | .001 |
|  |  | M3 vs M2a |  |  | | |  |  |  |  | .017 | .019 |
|  |  |  |  |  | | |  |  |  |  |  |  |
|  | T3 | M1 | 47.00 | 1.17 | | | .821 | .750 | .071 | .094 [.059, .128] |  |  |
|  |  | **M2a** | **26.93** | **1.10** | | | **.934** | **.892** | **.049** | **.060 [.000, .100]** |  |  |
|  |  | M2b | 48.29 | 1.13 | | | .806 | .714 | .070 | .098 [.064, .133] |  |  |
|  |  | M2c | 41.84 | 1.06 | | | .849 | .777 | .063 | .084 [.049, .119] |  |  |
|  |  | M3 | 35.75 | 1.17 | | | .889 | .837 | .059 | .076 [.035, .113] |  |  |
|  |  | M2a vs M1 |  |  | | |  |  |  |  | .113 | .034 |
|  |  | M2b vs M1 |  |  | | |  |  |  |  | .015 | .004 |
|  |  | M2c vs M1 |  |  | | |  |  |  |  | .028 | .010 |
|  |  | M3 vs M2a |  |  | | |  |  |  |  | .045 | .016 |
|  |  |  |  |  | | |  |  |  |  |  |  |
|  | T4 | M1 | 26.98 | 1.04 | | | .933 | .906 | .057 | .047 [.000, .088] |  |  |
|  |  | **M2a** | **8.91** | **1.02** | | | **1.000** | **1.000** | **.032** | **.000 [.000, .007]** |  |  |
|  |  | M2b | 28.57 | 0.98 | | | .908 | .864 | .057 | .055 [.000, .094] |  |  |
|  |  | M2c | 31.28 | 0.89 | | | .882 | .826 | .057 | .059 [.014, .095] |  |  |
|  |  | M3 | 9.23 | 0.84 | | | 1.000 | 1.000 | .030 | .000 [.000, .018] |  |  |
|  |  | M2a vs M1 |  |  | | |  |  |  |  | .067 | .047 |
|  |  | M2b vs M1 |  |  | | |  |  |  |  | .025 | .008 |
|  |  | M2c vs M1 |  |  | | |  |  |  |  | .051 | .012 |
|  |  | M3 vs M2a |  |  | | |  |  |  |  | .000 | .000 |
|  |  |  |  |  | | |  |  |  |  |  |  |
|  | T5 | M1 | 32.78 | 0.95 | | | .968 | .960 | .056 | .034 [.011, .070] |  |  |
|  |  | **M2a** | **20.07** | **1.19** | | | **.981** | **.972** | **.049** | **.018 [.000, .071]** |  |  |
|  |  | M2b | 20.23 | 0.95 | | | .978 | .967 | .049 | .020 [.000, .073] |  |  |
|  |  | M2c | 21.06 | 0.93 | | | .963 | .945 | .050 | .026 [.000, .075] |  |  |
|  |  | M3 | 20.80 | 0.89 | | | .931 | .887 | .048 | .036 [.000, .082] |  |  |
|  |  | M2a vs M1 |  |  | | |  |  |  |  | .013 | .016 |
|  |  | M2b vs M1 |  |  | | |  |  |  |  | .010 | .014 |
|  |  | M2c vs M1 |  |  | | |  |  |  |  | .005 | .008 |
|  |  | M3 vs M2a |  |  | | |  |  |  |  | .050 | .018 |

M1 = single-factor model; M2a = inhibition-switching and updating model; M2b = inhibition-updating and switching model; M2c = switching -updating and inhibition model; M3 = three-factor model. The *df*s for M1, M2a, M2b, M2c, and M3 were 20, 19, 19, 19, and 17, respectively.

**Table S10**

*The confirmatory factor analysis results of executive functions for cohort G5 at T2, G7 at T1, G9 at T4 and T5*

| Cohort | Wave | model | *χ^2^* | Scaling | CFI | TLI | SRMR | RMSEA (90% CI) | ΔCFI | ΔRMSEA |
| --- | --- | --- | --- | --- | --- | --- | --- | --- | --- | --- |
| G5 | T2 | M1 | 65.30 | 0.97 | .786 | .680 | .074 | .114 [.087, .142] |  |  |
|  |  | M2a | 21.39 | 0.99 | .965 | .944 | .039 | .048 [.000, .083] |  |  |
|  |  | M2b | 57.47 | 0.98 | .815 | .701 | .072 | .110 [.082, .139] |  |  |
|  |  | M2c | 54.04 | 0.94 | .829 | .724 | .067 | .105 [.077, .135] |  |  |
|  |  | **M3** | **10.27** | **0.95** | **1.000** | **1.006** | **.025** | **.000 [.000, .059]** |  |  |
|  |  | M2a vs M1 |  |  |  |  |  |  | .179 | .066 |
|  |  | M2b vs M1 |  |  |  |  |  |  | .029 | .004 |
|  |  | M2c vs M1 |  |  |  |  |  |  | .043 | .009 |
|  |  | M3 vs M2a |  |  |  |  |  |  | .035 | .048 |
|  |  |  |  |  |  |  |  |  |  |  |
| G7 | T1 | M1 | 46.39 | 0.85 | .689 | .533 | .089 | .137 [.095, .182] |  |  |
|  |  | M2a | 29.81 | 0.89 | .838 | .739 | .073 | .103 [.054, .151] |  |  |
|  |  | M2b | 50.82 | 0.84 | .705 | .566 | .086 | .117 [.078, .156] |  |  |
|  |  | M2c | 23.48 | 0.89 | .899 | .837 | .059 | .081 [.020, .133] |  |  |
|  |  | **M3** | **13.52** | **0.93** | **.976** | **.954** | **.040** | **.043 [.000, .109]** |  |  |
|  |  | M2a vs M1 |  |  |  |  |  |  | .149 | .034 |
|  |  | M2b vs M1 |  |  |  |  |  |  | .016 | .020 |
|  |  | M2c vs M1 |  |  |  |  |  |  | .210 | .056 |
|  |  | M3 vs M2c |  |  |  |  |  |  | .077 | .038 |
|  |  |  |  |  |  |  |  |  |  |  |
| G9 | T4 | M1 | 29.07 | 0.97 | .853 | .779 | .064 | .079 [.037, .119] |  |  |
|  |  | **M2a** | **4.74** | **0.98** | **1.000** | **1.130** | **.021** | **.000 [.000, .000]** |  |  |
|  |  | M2b | 32.01 | 0.88 | .814 | .700 | .064 | .092 [.052, .132] |  |  |
|  |  | M2c | 36.01 | 0.78 | .775 | .637 | .063 | .101 [.062, .141] |  |  |
|  |  | M3 | 15.57 | 0.89 | 1.000 | 1.036 | .050 | .000 [.000, .000] |  |  |
|  |  | M2a vs M1 |  |  |  |  |  |  | .147 | .079 |
|  |  | M2b vs M1 |  |  |  |  |  |  | .039 | .013 |
|  |  | M2c vs M1 |  |  |  |  |  |  | .078 | .022 |
|  |  | M3 vs M2a |  |  |  |  |  |  | .000 | .000 |
|  |  |  |  |  |  |  |  |  |  |  |
|  | T5 | M1 | 14.97 | 0.94 | .981 | .972 | .043 | .021 [.000, .080] |  |  |
|  |  | **M2a** | **13.42** | **0.92** | **.992** | **.987** | **.040** | **.014 [.000, .080]** |  |  |
|  |  | M2b | 15.01 | 0.94 | .961 | .937 | .043 | .031 [.000, .087] |  |  |
|  |  | M2c^*^ | **-** | **-** | **-** | **-** | **-** | **-** |  |  |
|  |  | M3^*^ | **-** | **-** | **-** | **-** | **-** | **-** |  |  |
|  |  | M2a vs M1 |  |  |  |  |  |  | .011 | .007 |
|  |  | M2b vs M1 |  |  |  |  |  |  | .020 | .010 |

M1 = single-factor model; M2a = inhibition-switching and updating model; M2b = inhibition-updating and switching model; M2c = switching -updating and inhibition model; M3 = three-factor model. The *df*s for M1, M2a, M2b, M2c, and M3 were 14, 13, 13, 13, and 11, respectively.

^*^The standard errors of the model parameter estimates could not be computed.

**Fig S1**

*Diagrams of factor models*

**
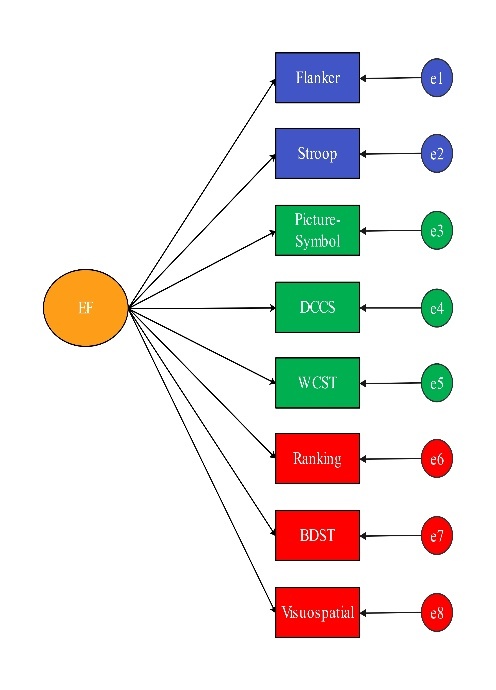

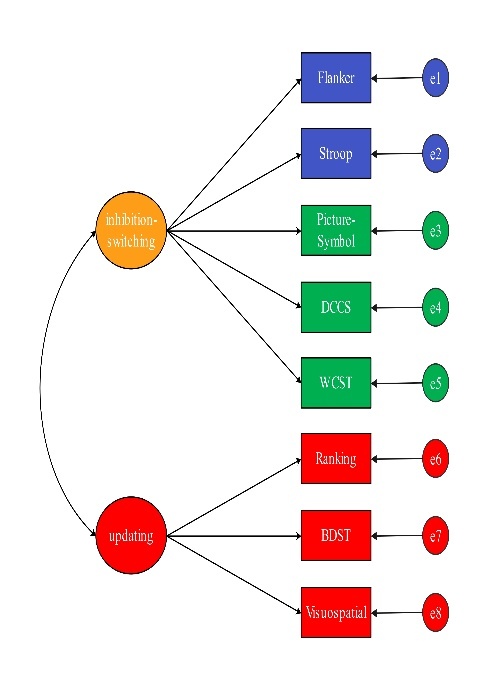

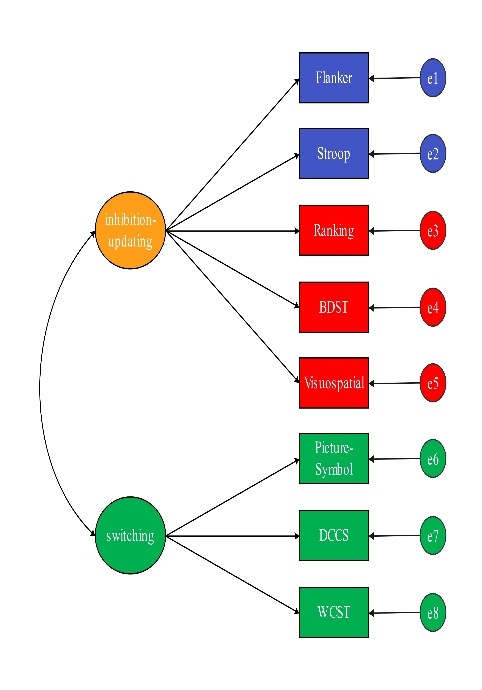

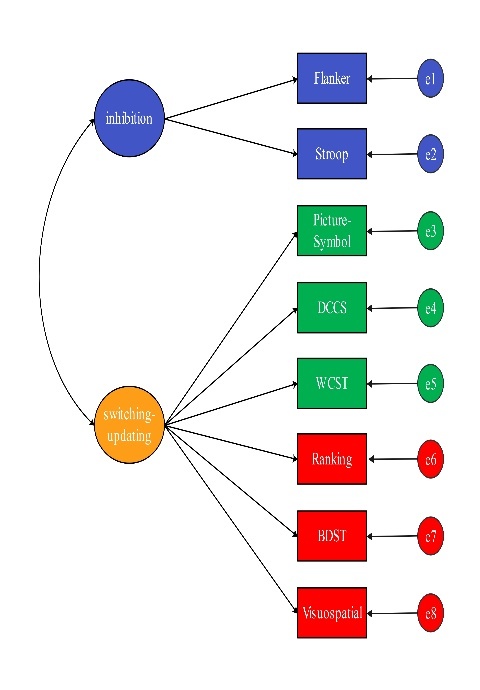

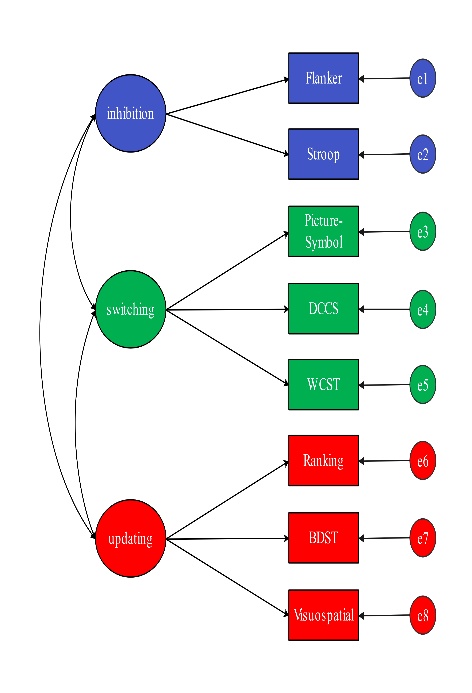
**

M1 M2a M2b M2c M3

**Fig S2**

*Age-related differences in mean performance on the inhibition, switch, and updating tasks*

**Fig S3**

*EF component structure across cohorts and waves (multiple imputation)*

|  | T1 | T2 | T3 | T4 | T5 |
| --- | --- | --- | --- | --- | --- |
| Cohort G5 | 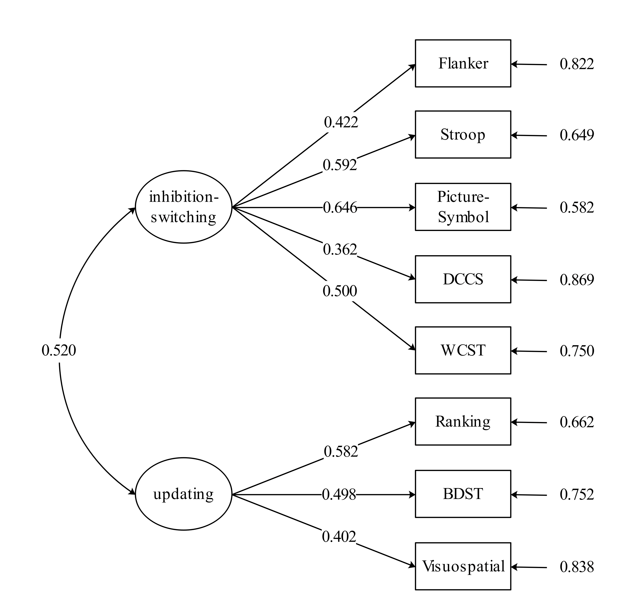 | 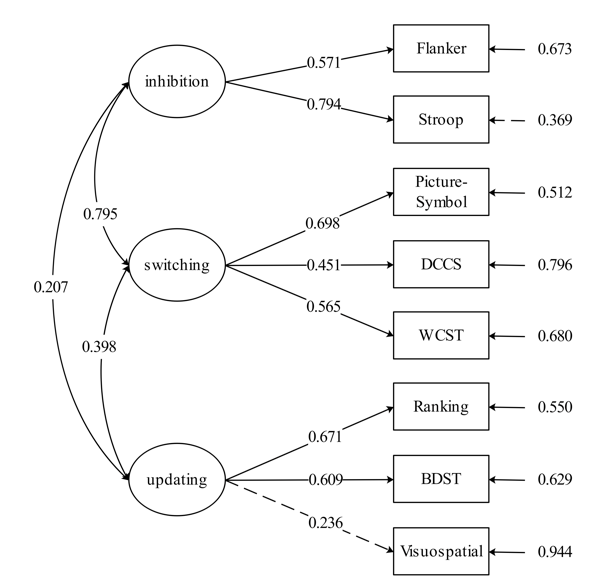 | 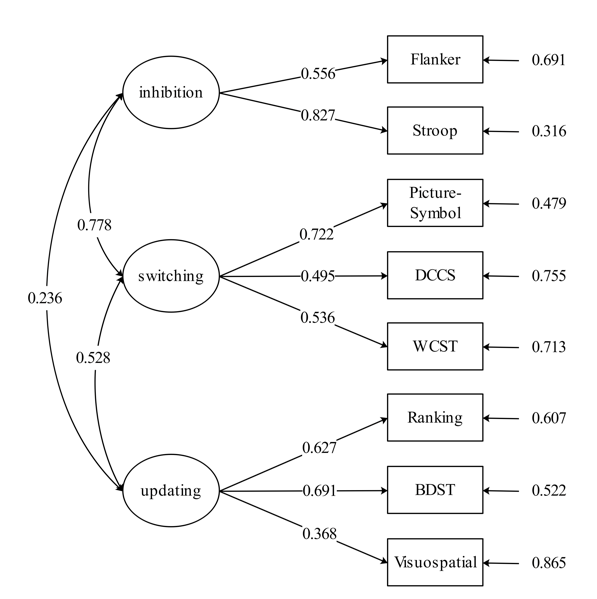 | 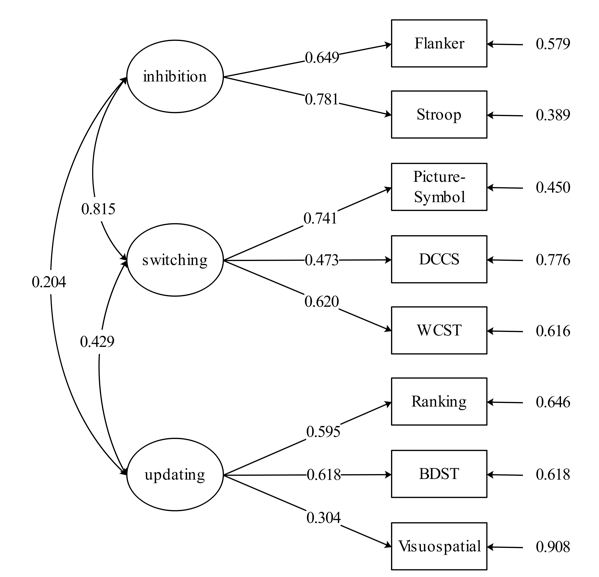 | 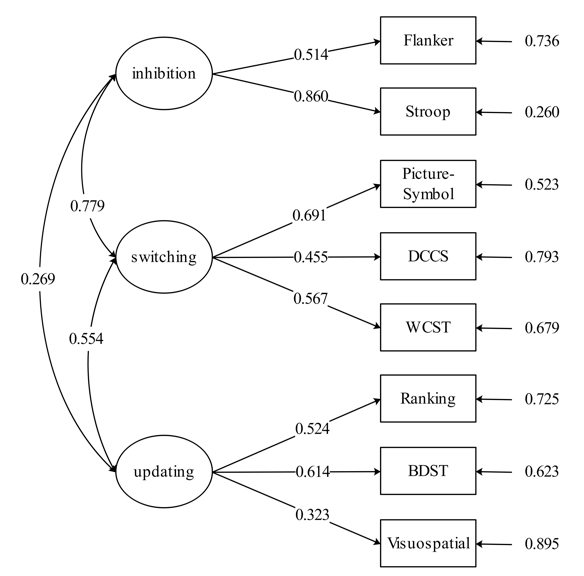 |
| Cohort G7 | 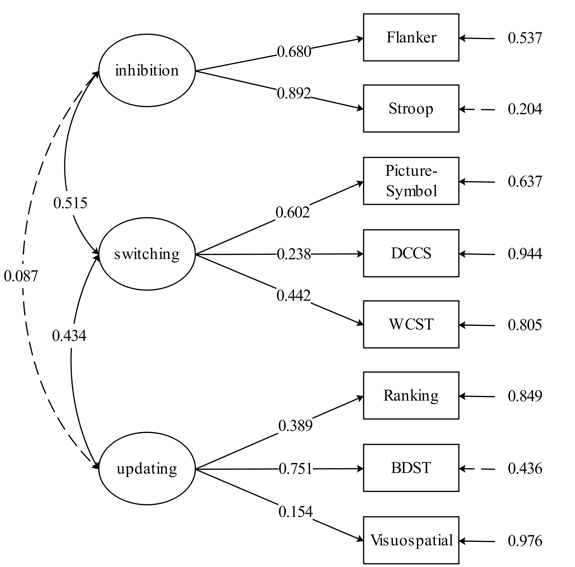 | 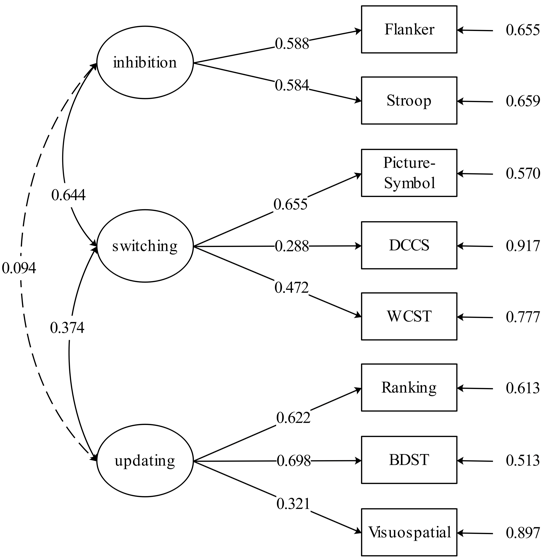 | 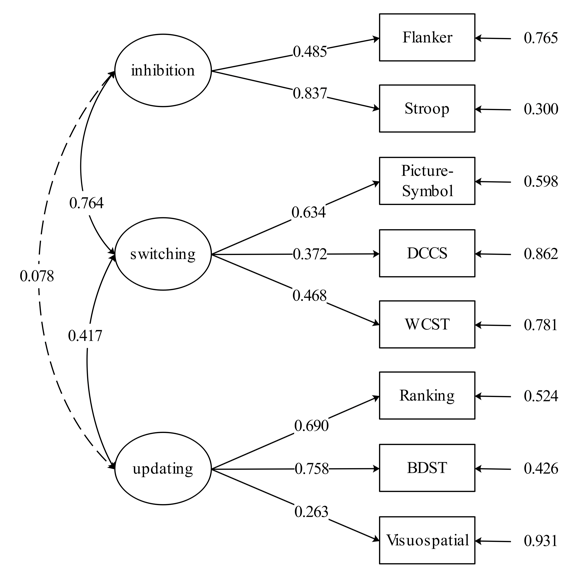 | 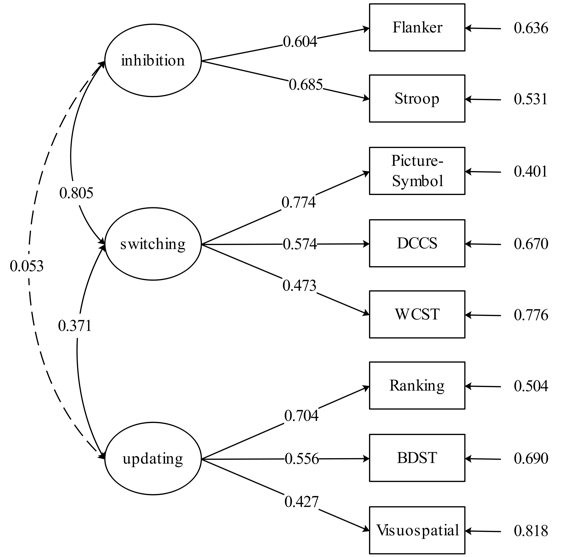 | 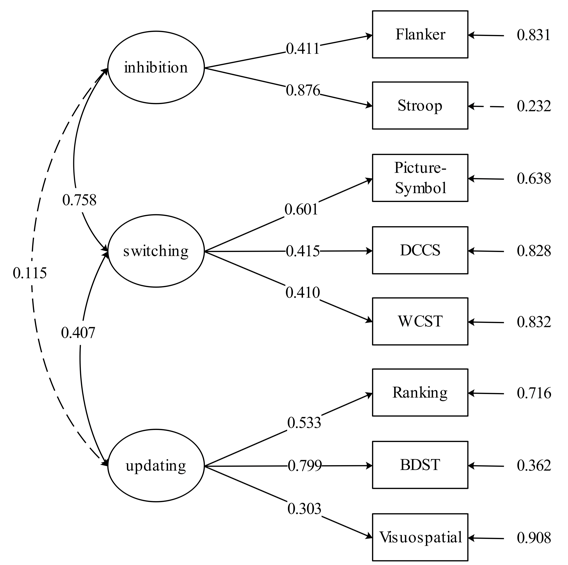 |
| Cohort G9 | 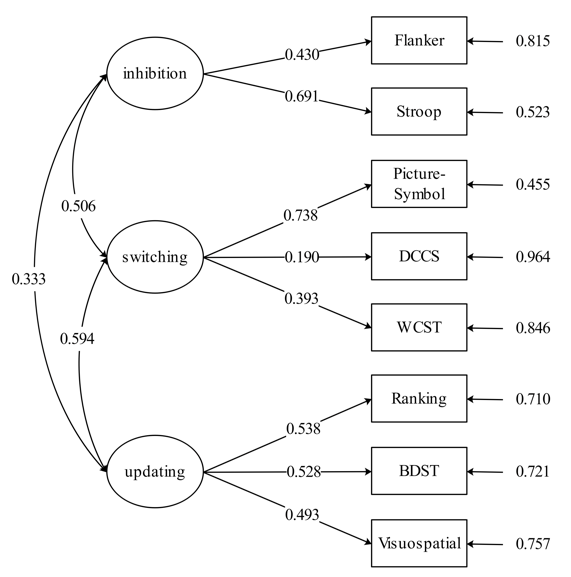 | 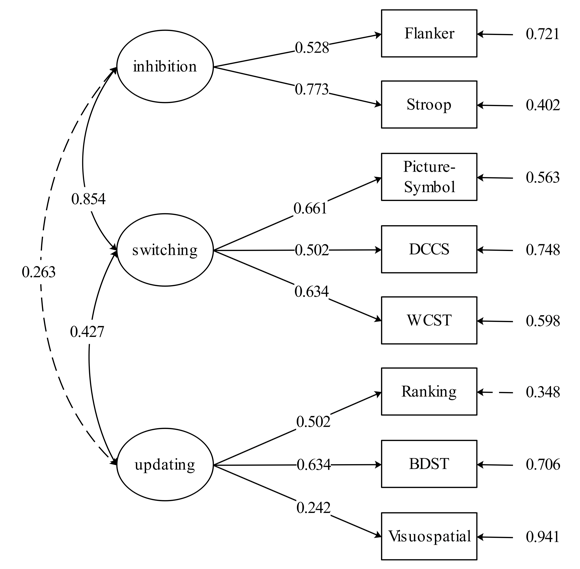 | 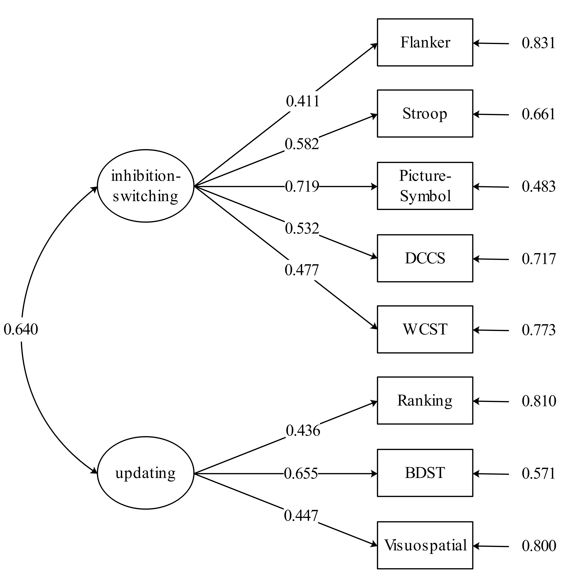 | 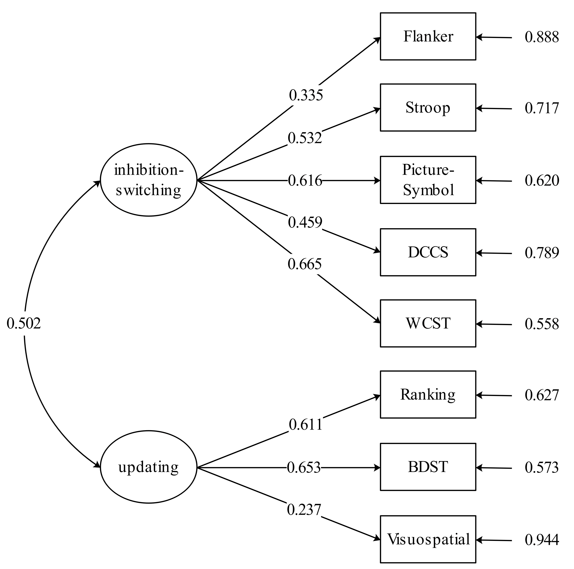 | 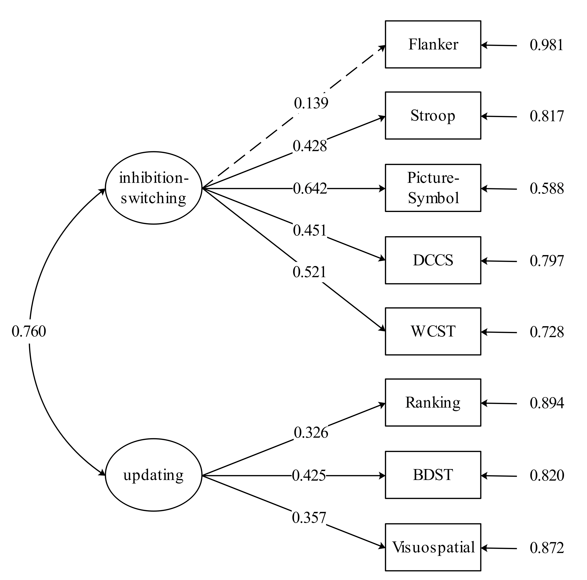 |

**Fig S4**

*Participant-level distributions of task*


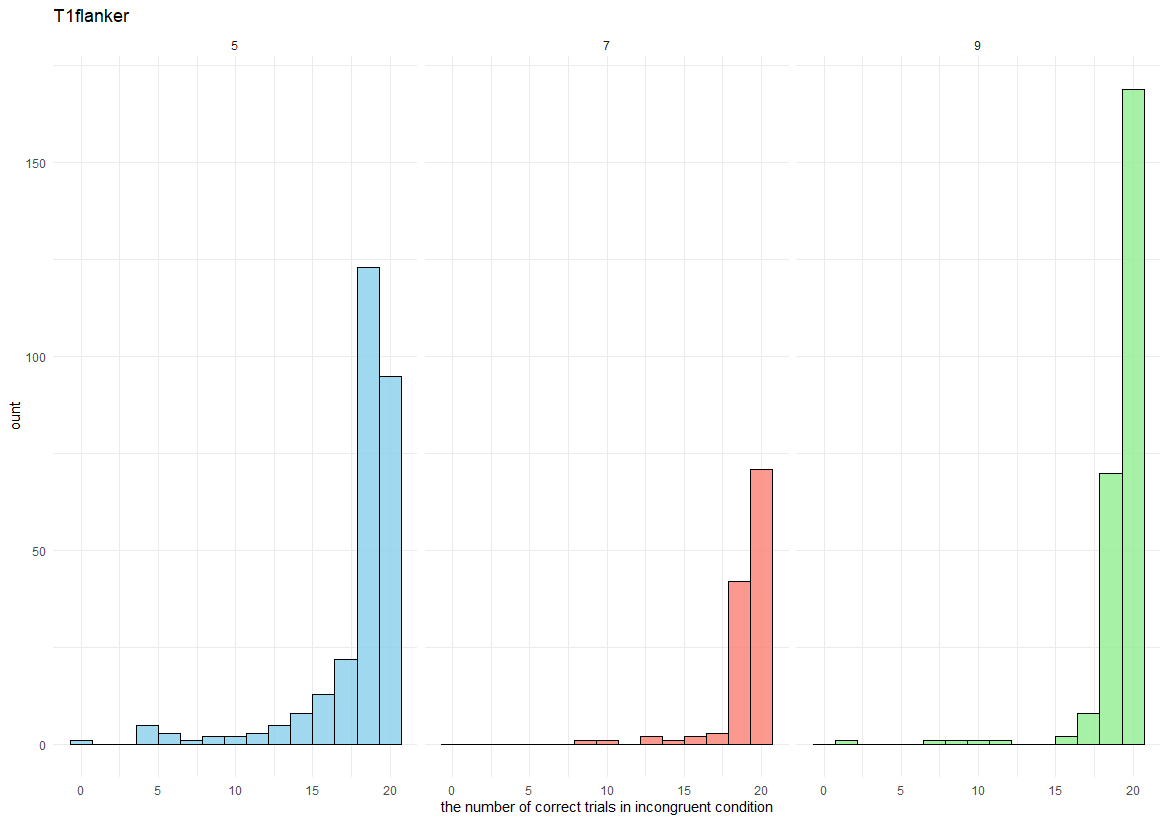

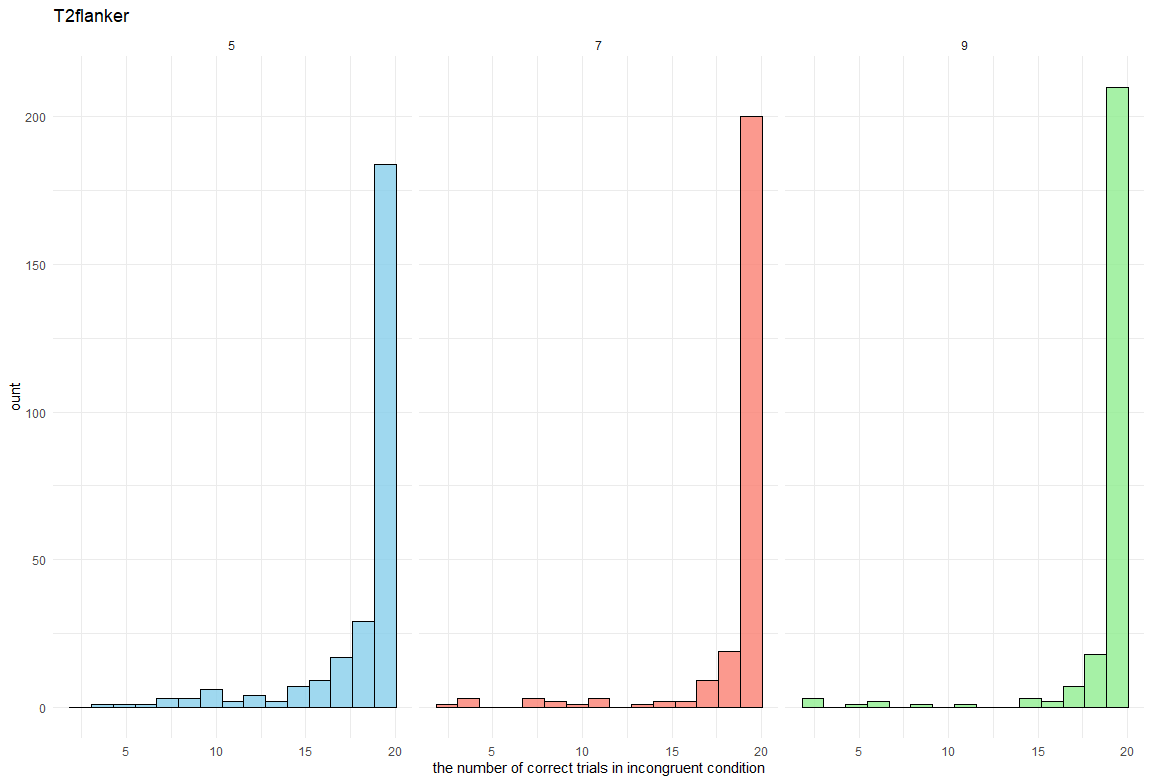

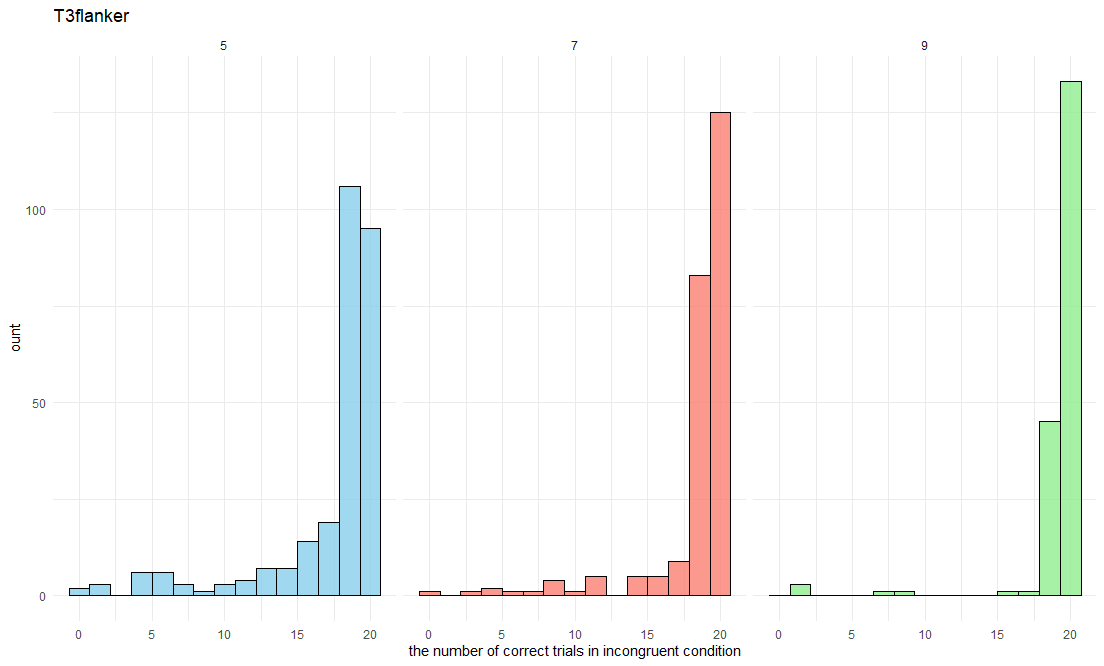

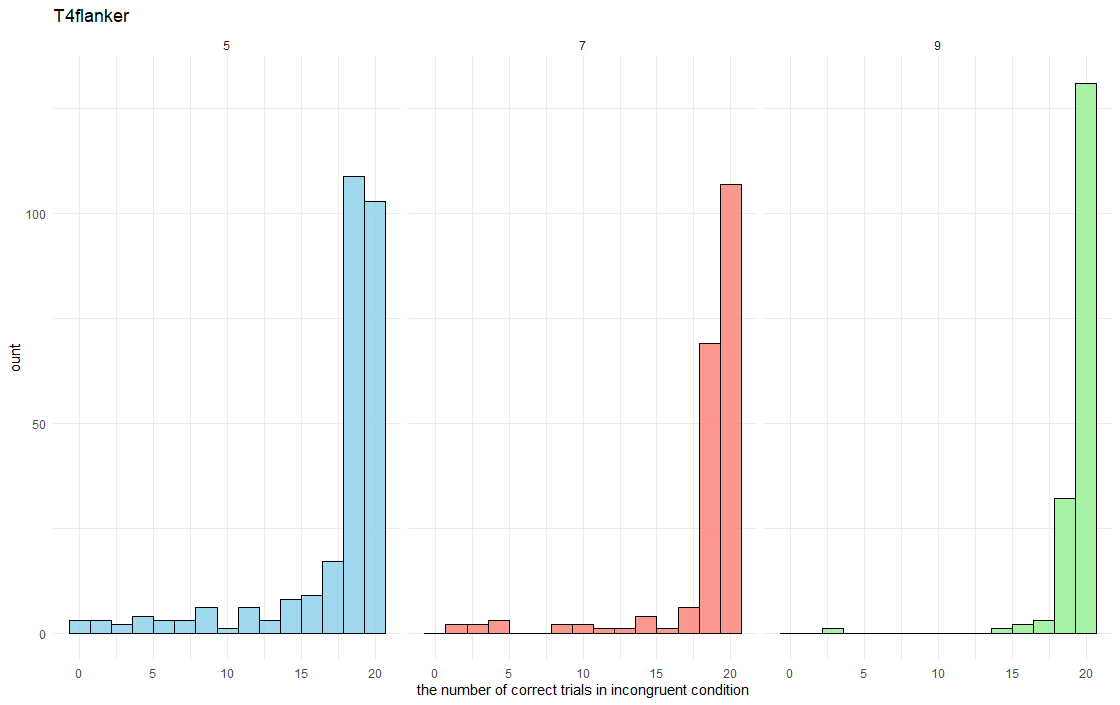

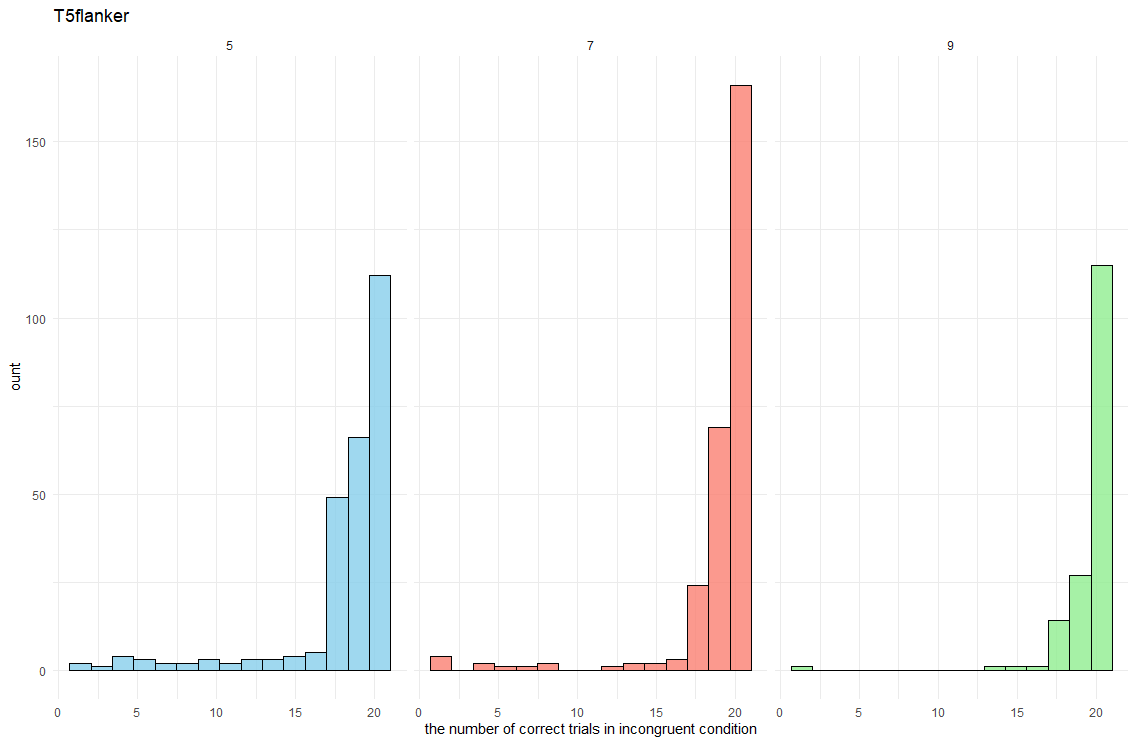


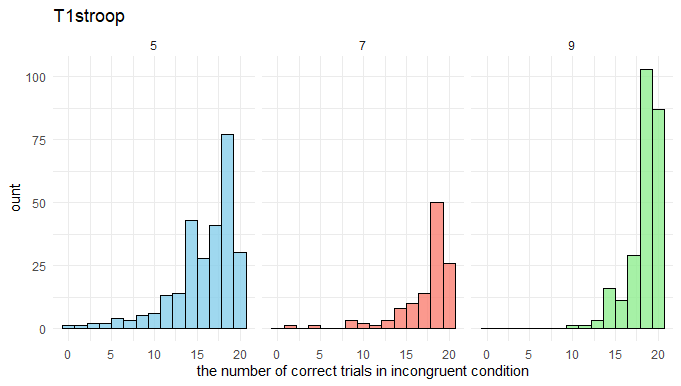

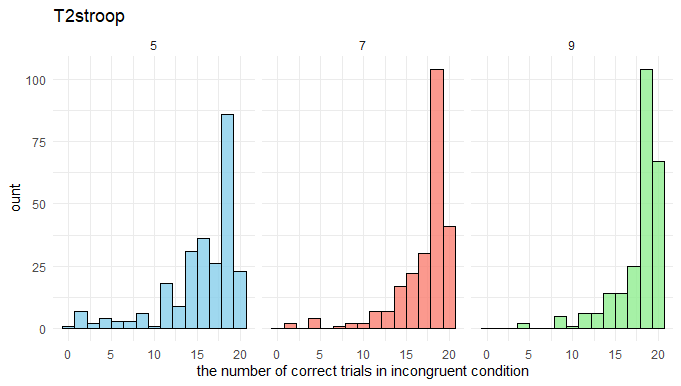

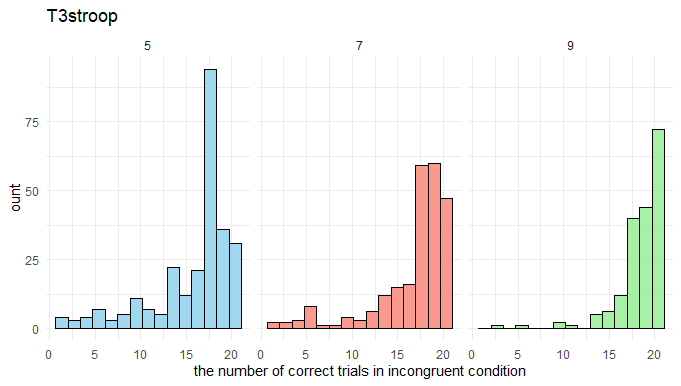

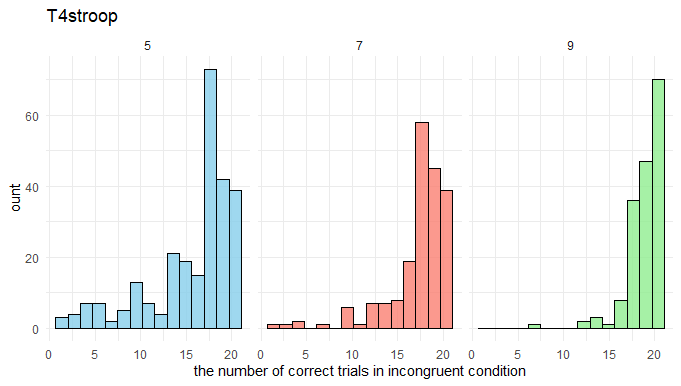

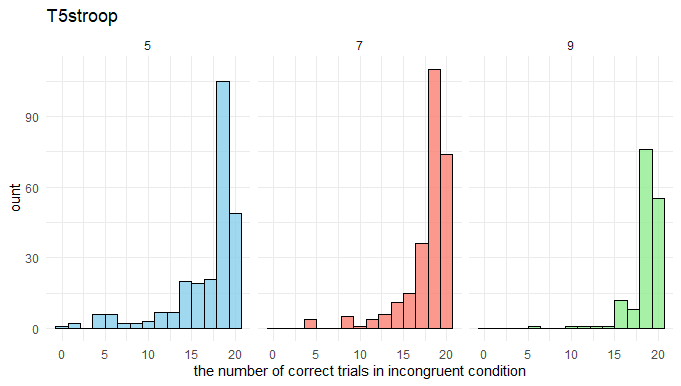


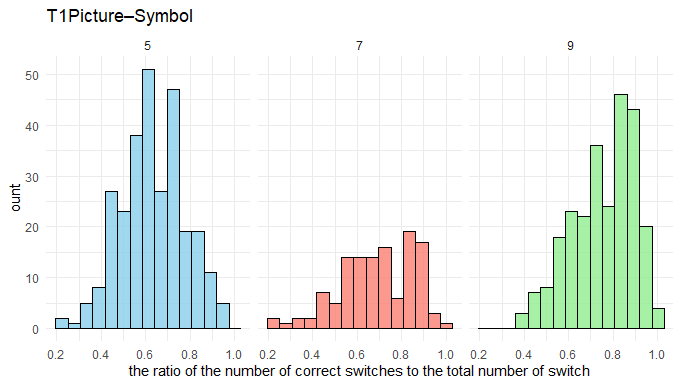

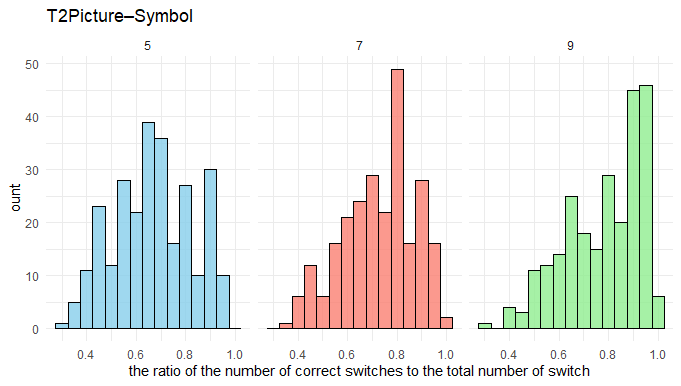

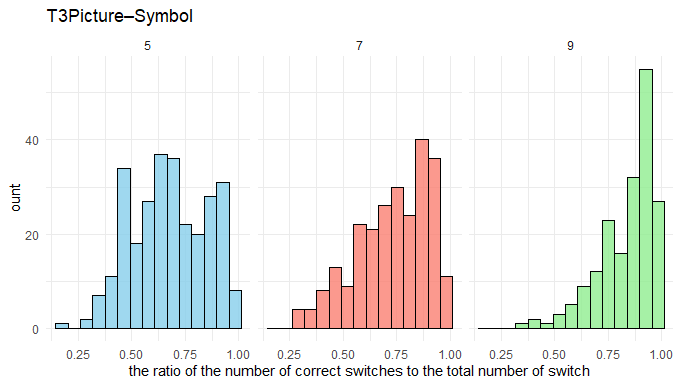

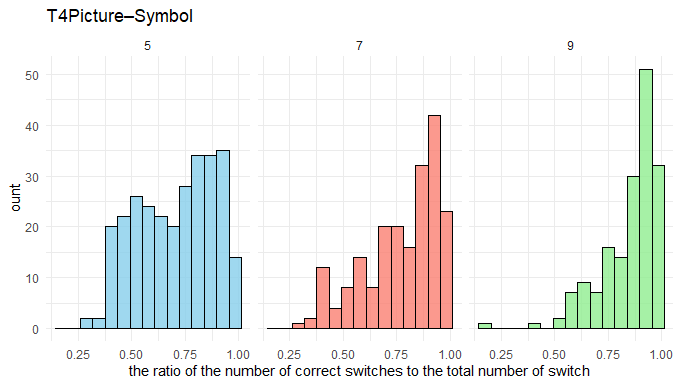

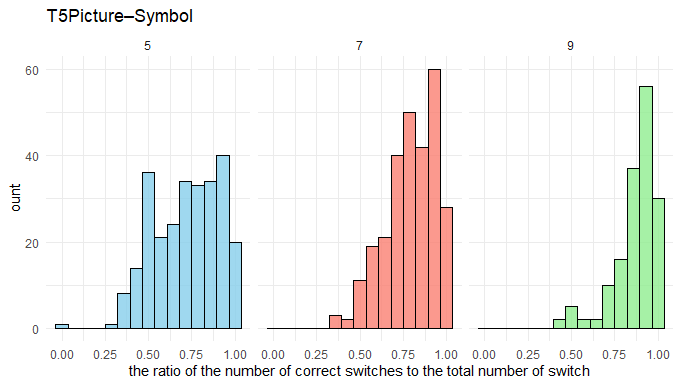


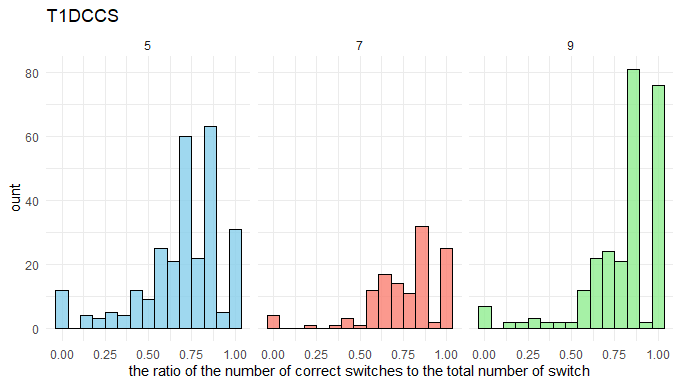

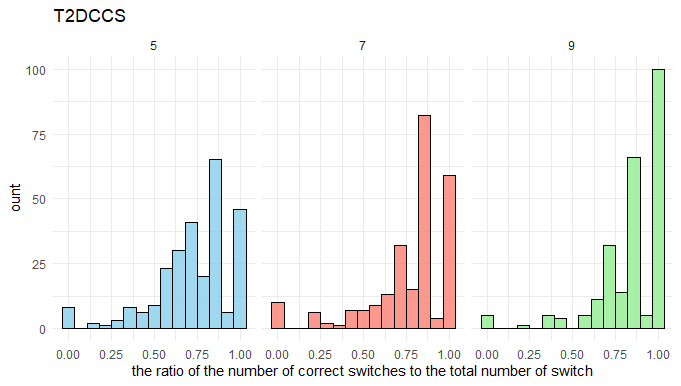

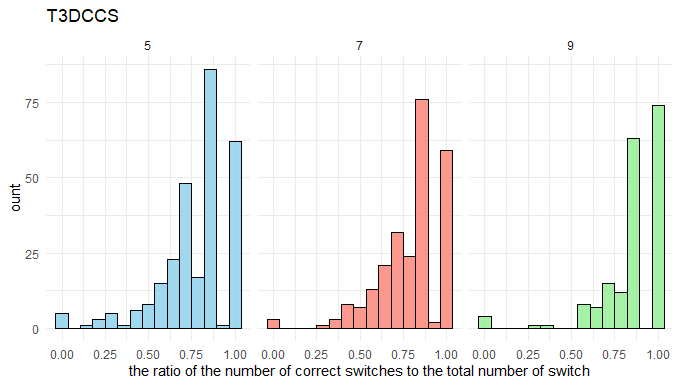

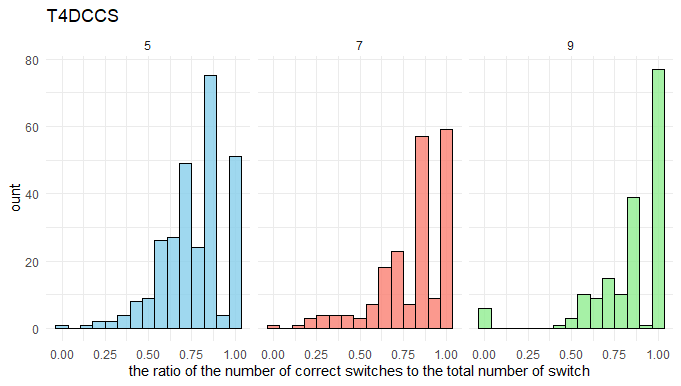

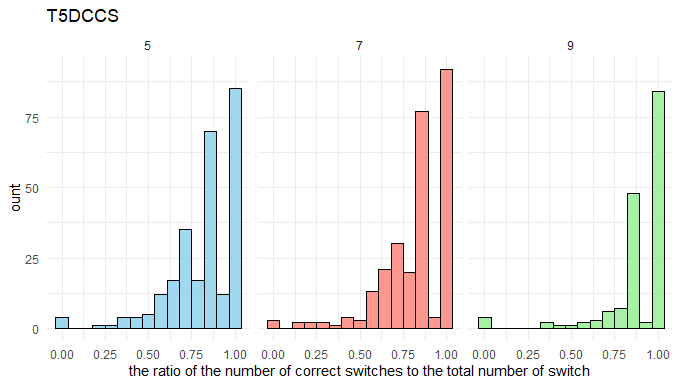


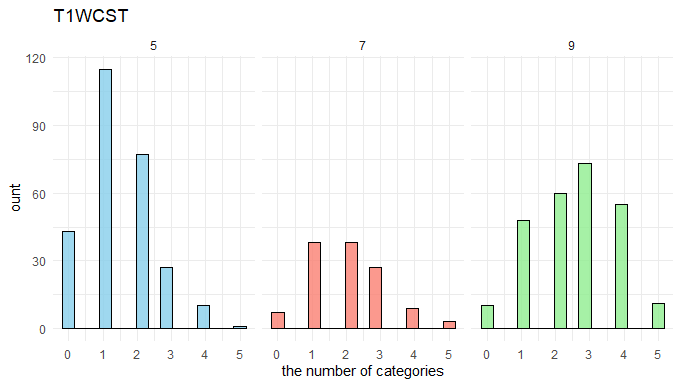

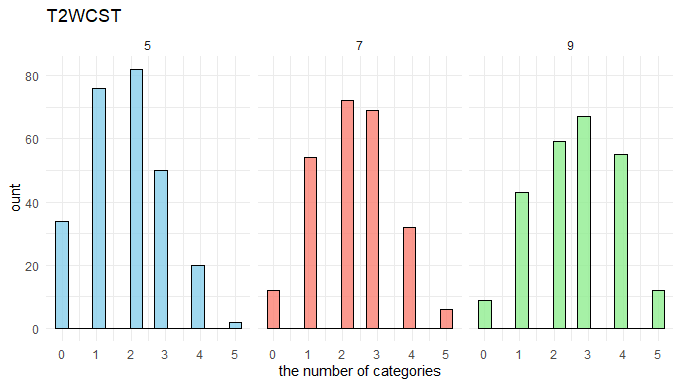

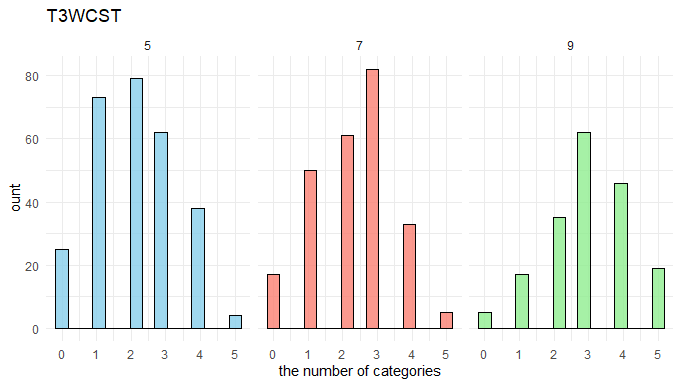

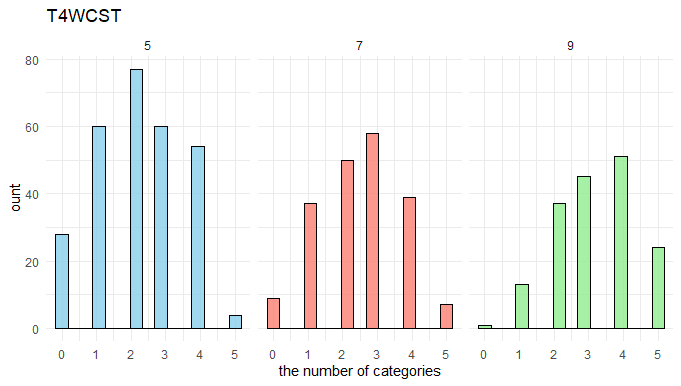

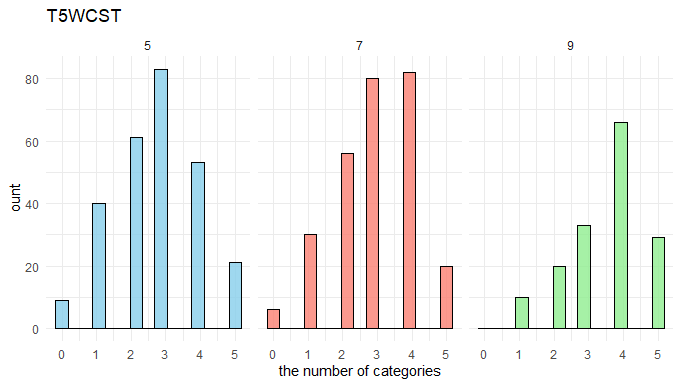


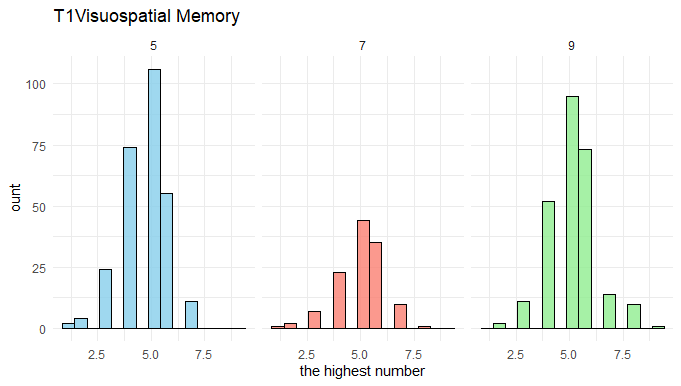

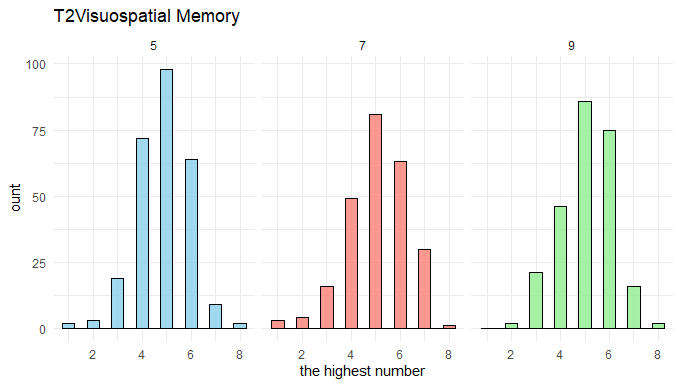

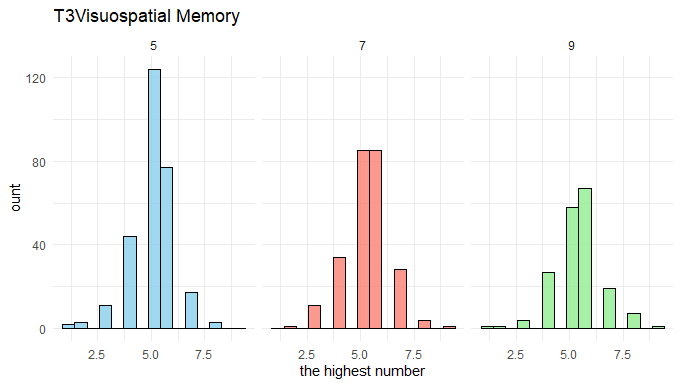

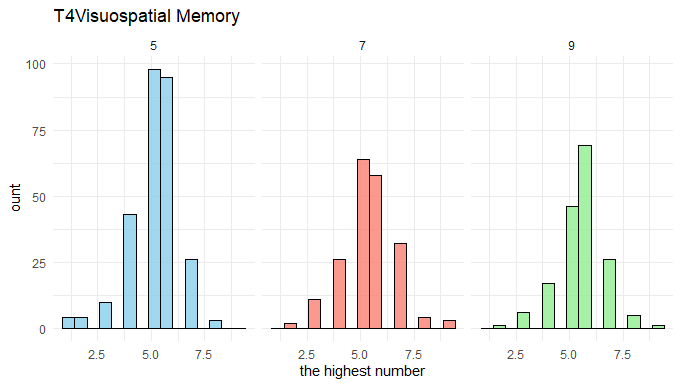

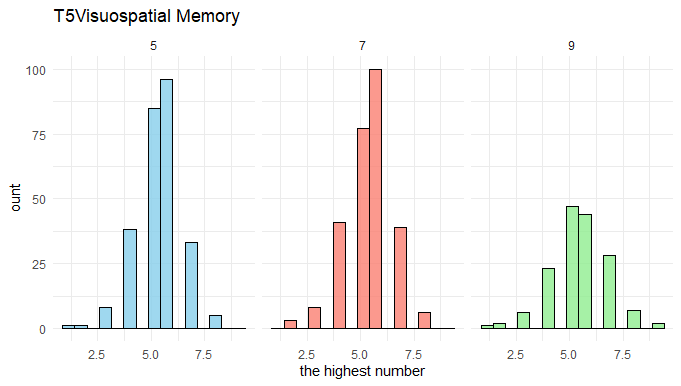


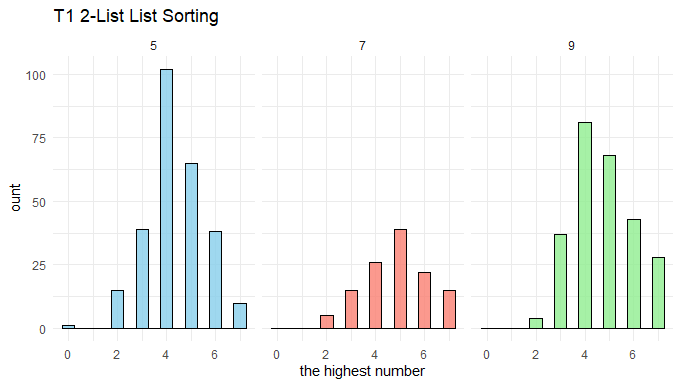

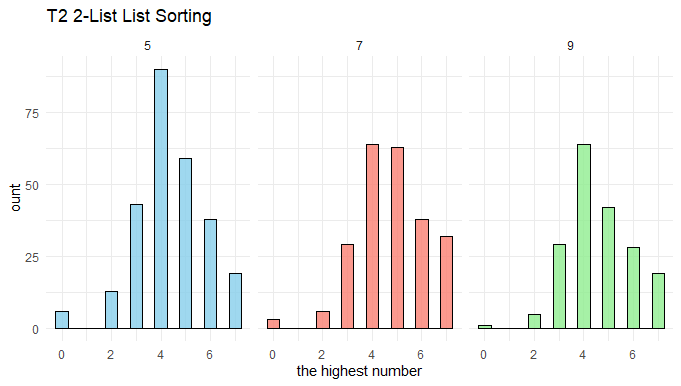

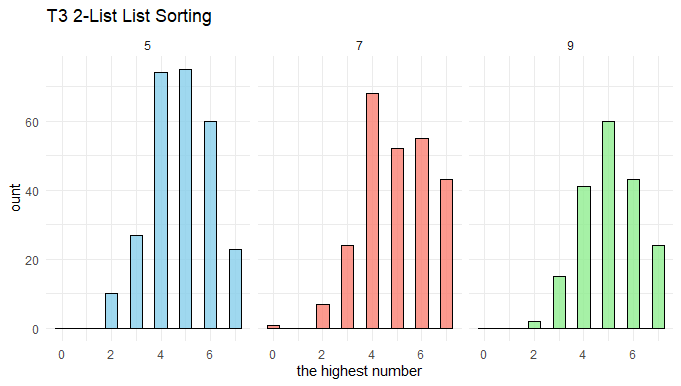

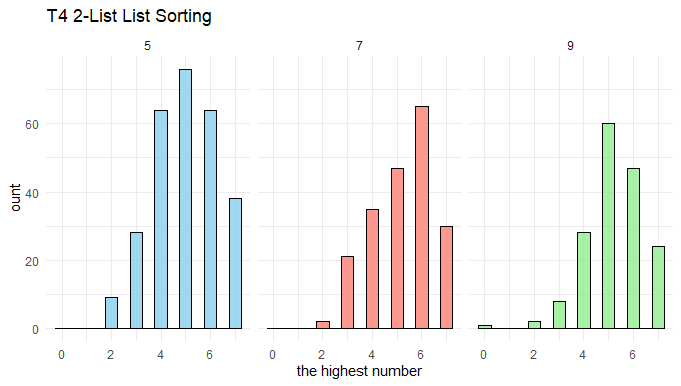

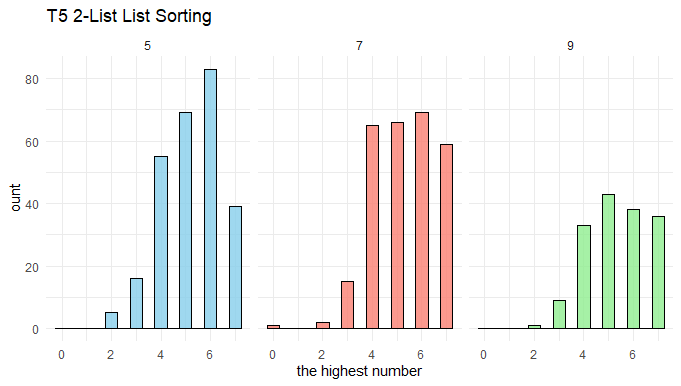


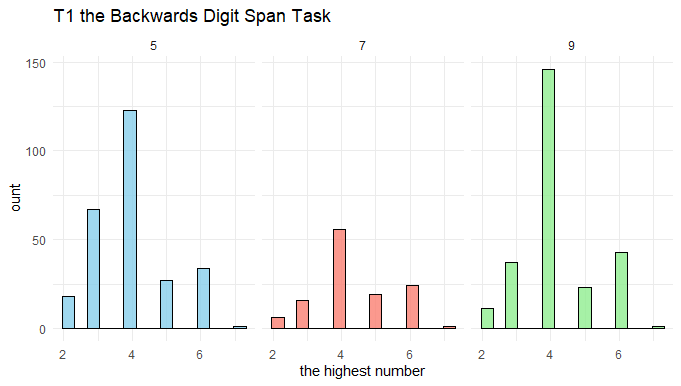

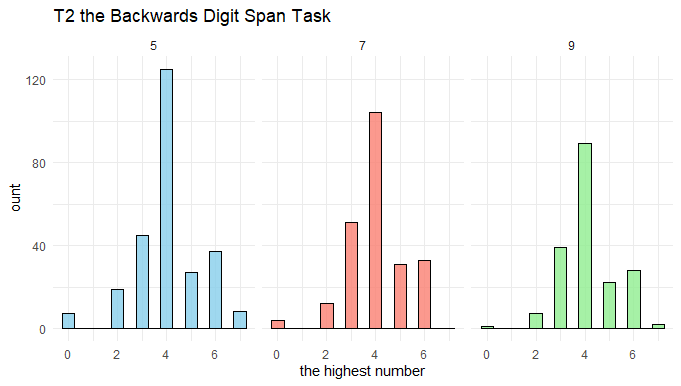

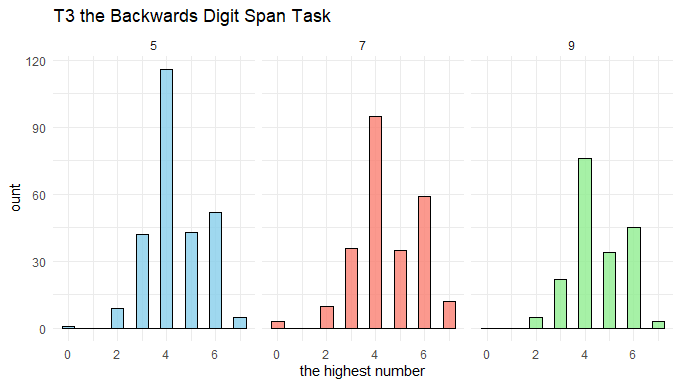

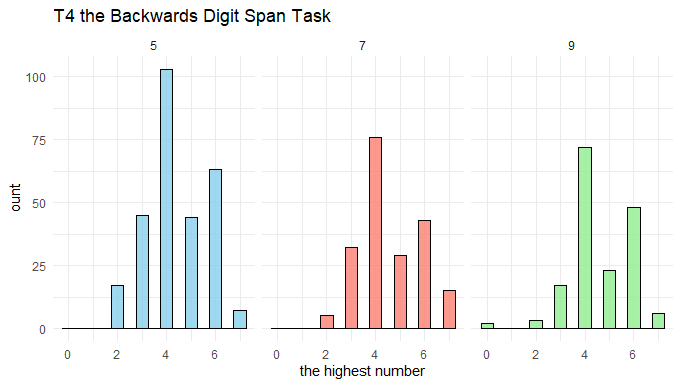

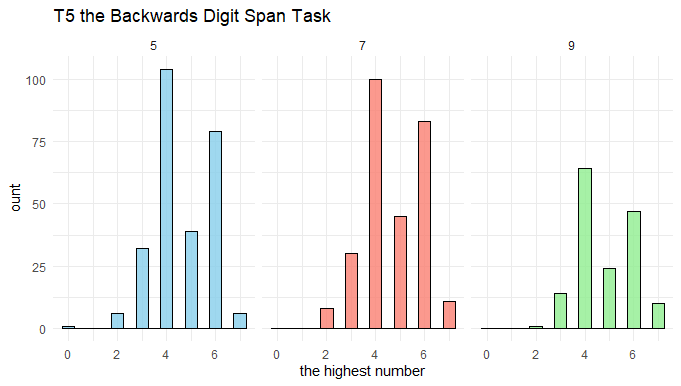

Supplement: Supplementary file 1 — Supplementary Material 1 [file 10964_2025_2293_MOESM1_ESM.docx]
